# A bioenergy-focused versus a reforestation-focused mitigation pathway yields disparate carbon storage and climate responses

**DOI:** 10.1073/pnas.2306775121

**Published:** 2024-02-05

**Authors:** Yanyan Cheng, David M. Lawrence, Ming Pan, Baoqing Zhang, Neal T. Graham, Peter J. Lawrence, Zhongfang Liu, Xiaogang He

**Affiliations:** ^a^Department of Industrial Systems Engineering and Management, National University of Singapore, 117576, Singapore; ^b^Climate and Global Dynamics Laboratory, National Science Foundation National Center for Atmospheric Research, Boulder, CO 80305; ^c^Center for Western Weather and Water Extremes, Scripps Institution of Oceanography, University of California San Diego, La Jolla, CA 92093; ^d^Key Laboratory of Western China’s Environmental Systems (Ministry of Education), College of Earth and Environmental Sciences, Lanzhou University, Lanzhou, Gansu 730000, China; ^e^Joint Global Change Research Institute, Pacific Northwest National Laboratory, College Park, MD 20740; ^f^State Key Laboratory of Marine Geology, Tongji University, Shanghai 200092, China; ^g^Department of Civil and Environmental Engineering, National University of Singapore, 117576, Singapore

**Keywords:** BECCS, reforestation, bioenergy, climate mitigation, carbon dioxide removal

## Abstract

Limiting global warming to 2 °C requires urgent action on land-based mitigation. This study evaluates the biogeochemical and biogeophysical implications of two alternative land-based mitigation scenarios that aim to achieve the same radiative forcing. One scenario is primarily driven by bioenergy expansion (SSP226Lu-BIOCROP), while the other involves re/afforestation (SSP126Lu-REFOREST). We find that overall, SSP126Lu-REFOREST is a more efficient strategy for removing CO_2_ from the atmosphere by 2100, resulting in a net carbon sink of 242 ~ 483 PgC with smaller uncertainties compared to SSP226Lu-BIOCROP, which exhibits a wider range of −78 ~ 621 PgC. However, SSP126Lu-REFOREST leads to a relatively warmer planetary climate than SSP226Lu-BIOCROP, and this relative warming can be intensified in certain re/afforested regions where local climates are not favorable for tree growth. Despite the cooling effect on a global scale, SSP226Lu-BIOCROP reshuffles regional warming hotspots, amplifying summer temperatures in vulnerable tropical regions such as Central Africa and Southeast Asia. Our findings highlight the need for strategic land use planning to identify suitable regions for re/afforestation and bioenergy expansion, thereby improving the likelihood of achieving the intended climate mitigation outcomes.

## Significance

Our study compares two land-based mitigation scenarios that limit global warming to 2 °C, primarily driven by bioenergy expansion and re/afforestation. While the re/afforestation-focused scenario excels in CO_2_ removal with lower uncertainties, it could lead to a relatively warmer regional climate than the bioenergy expansion–focused scenario, especially in regions unfavorable for tree growth. Despite the global cooling effect, the bioenergy expansion–focused approach, however, reshuffles regional warming hotspots, potentially amplifying summer temperatures in vulnerable regions such as Central Africa and Southeast Asia. Our study highlights the importance of carefully locating suitable re/afforestation and bioenergy expansion regions for achieving intended climate mitigation outcomes. Our research provides valuable insights for future land use planning and policy decisions to mitigate climate change.

Future Shared Socioeconomic Pathways (SSPs) that aim to achieve greenhouse gas levels commensurate with a 2 °C warming target require substantial land-based greenhouse gas mitigation strategies, such as bioenergy with carbon capture and storage (BECCS) and/or reforestation, afforestation, and forest restoration (1–7). The IPCC’s Sixth Assessment Report (AR6) projects a 2.4 ~ 10.9 million km^2^ of bioenergy crop deployment over the next 80 y in the SSP2-2.6 scenario (8) (i.e., the SSP2 “middle of the road” pathway combined with the Representative Concentration Pathway 2.6, RCP2.6) (9). Compared to bioenergy expansion, the alternative re/afforestation-based mitigation strategy (SSP1-2.6) requires up to 9.6 million km^2^ of re/afforested areas by 2100 to achieve the same end-of-the-century radiative forcing (10) (e.g., RCP2.6, stabilizing global radiative forcing at 2.6 W/m^2^ or limiting warming below 2 °C by 2100). The key assumption underlying the SSP framework (9, 11) is that multiple socioeconomic scenarios can achieve the same radiative forcing and deliver similar global climate outcomes (12, 13). However, divergent land-use change patterns in alternative SSPs may challenge this assumption (12), especially when regional and seasonal heterogeneities of land climate outcomes are considered.

Meanwhile, growing evidence shows that there can be unintended consequences from large-scale bioenergy expansion including bioenergy cultivation–induced carbon emissions (2, 14–16), exacerbated water stress (16–19), and additional greenhouse gas emissions (20) resulting from irrigation and fertilizer application, and regional warming and drying due to bioenergy expansion–induced deforestation (21). Such costs may even exceed the projected carbon removal benefits of BECCS (2). However, existing assessments of BECCS either focus on the effectiveness of carbon removal (2) or biophysically-driven climate change impacts (21, 22). Given that biogeochemical and biogeophysical outcomes can diverge, a consensus accounting of the two effects is yet to be done.

In addition, the net biogeophysical effects of re/afforestation on climate are complex and differ across regions due to varying influences of albedo, surface roughness, and evapotranspiration (ET) (23, 24). While tropical forests are demonstrated to be climate coolers, re/afforestation in boreal regions could result in warming due to the snow-vegetation albedo feedback (23). In some regions, the biogeophysical response to re/afforestation can fully offset the climate benefits from forest carbon sequestration. For example, none of the proposed European forest management scenarios appear to be effective at reducing local climate warming (25). Therefore, it is critical to identify locations that can maximize re/afforestation-based mitigation potential (23) and such evaluations benefit from assessment in a fully coupled Earth system model (ESM) that can capture land-atmosphere feedback.

In this study, we apply an integrated human–Earth system modeling framework (26), GCAM-Demeter-CESM_bioenergy_ (27–29) (*Methods*), to investigate the impact of alternative land-based mitigation pathways that focus on either bioenergy expansion or re/afforestation on land carbon sink and climate. A fully coupled ESM rather than a land-only model (16) is necessary for our study, as it allows us to capture the dynamic, multi-scale (e.g., local-to-regional-to-global), and two-way land-atmosphere interactions (30), especially how land use changes feedback to the climate. We run two GCAM co-developed scenarios (SSP226Lu-BIOCROP, bioenergy expansion–focused; SSP126Lu-REFOREST, re/afforestation-focused) with contrasting land use pathways (SSP226Lu and SSP126Lu, *SI Appendix*, Figs. S1 and S2) that are designed to achieve the same radiative forcing level (RCP2.6). These experiments allow us to directly evaluate whether the biogeochemical and biogeophysical outcomes of alternative scenarios with divergent land-use changes comply with the SSP assumptions that were used to design them.

## Results

### Effectiveness of Re/Afforestation.

**Fig. 1.**
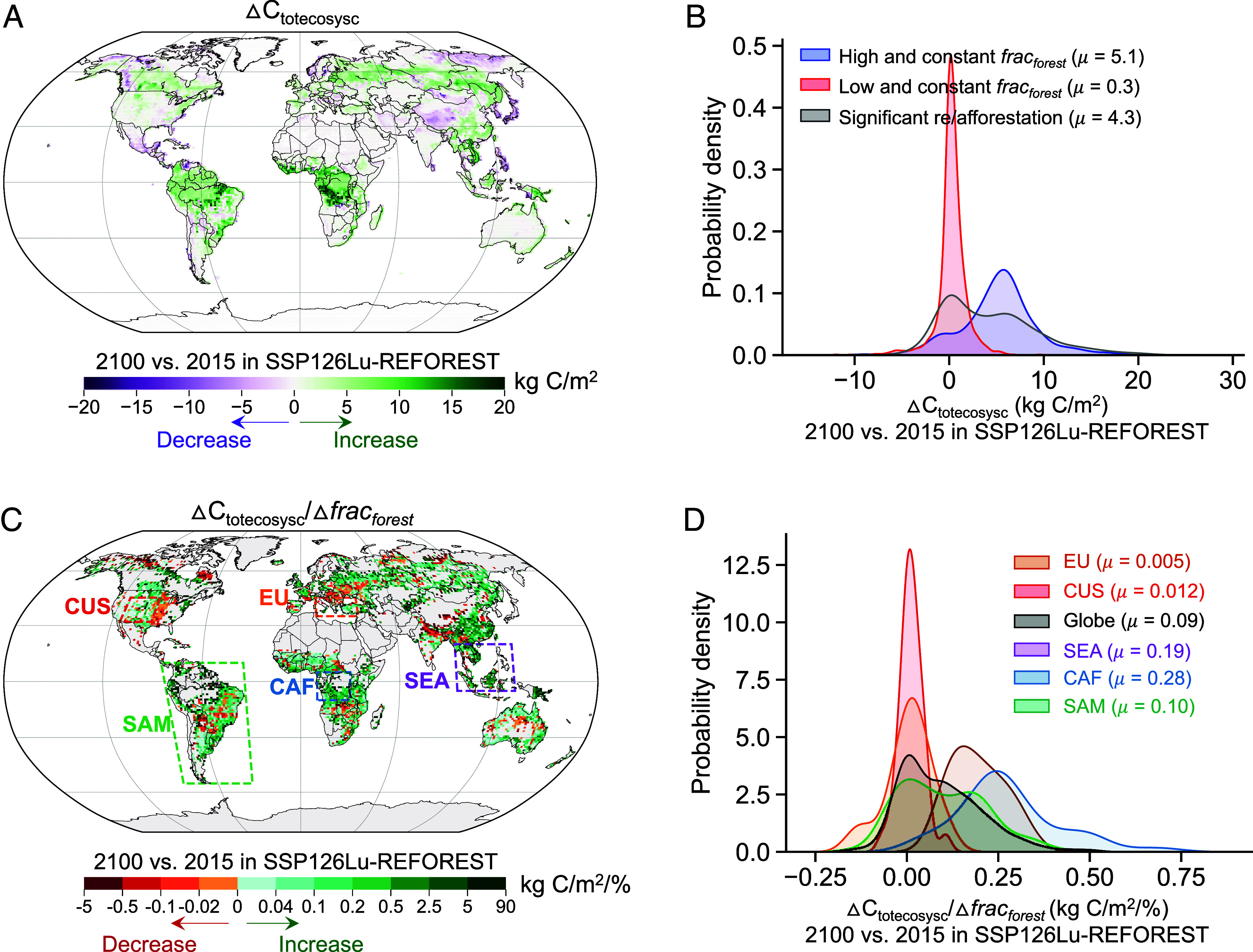
Changes in total ecosystem carbon under different types of land use changes and in successful/unsuccessful forest growth regions in the primary re/afforestation scenario. (*A*) Changes in total ecosystem carbon (ΔC_totecosysc_) between 2100 and 2015 (2100 minus 2015) in the primary re/afforestation scenario (SSP126Lu-REFOREST). (*B*) Distributions of ΔC_totecosysc_ for the grid cells with a high and constant forest fraction (blue color), a low and constant forest fraction (red color), and significant re/afforestation (gray color). (*C*) Changes in total ecosystem carbon normalized by changes in forest fraction (ΔCtotecosysc/Δfracforest) between 2100 and 2015 (2100 minus 2015) in SSP126Lu-REFOREST. (*D*) Distributions of ΔC_totecosysc_/Δ*frac_forest_* in grid cells with significant re/afforestation, averaged over globe (red color), two unsuccessful forest growth regions, which are CUS (red color) and EU (orange color), and three successful forest growth regions, which are SEA (purple color), CAF (blue color), and SAM (green color). The μ values in (*B* and *D*) are averaged ΔC_totecosysc_ and ΔC_totecosysc_/Δ*frac_forest_*, respectively.

We find that significant re/afforested areas (see *Methods* for definition) have a strong capacity to accumulate carbon although the capacity is somewhat lower than that of undisturbed forests (Fig. 1). We examine total ecosystem carbon gains from 2015 to 2100 (ΔC_totecosysc_ = C_totecosysc, 2100_ − C_totecosysc, 2015_, Fig. 1*A*) in SSP126Lu-REFOREST for grid cells that retain a high or a low forest cover throughout the future period against grid cells that undergo significant re/afforestation (*SI Appendix*, Fig. S3). The mean values of ΔC_totecosysc_ for the three types of grid cells are 5.1, 0.3, and 4.3 kg C/m^2^, respectively (Fig. 1*B*). As expected, areas covered with initial high forest fractions gain a large amount of carbon (*SI Appendix*, Fig. S5*A*), likely due to the CO_2_ fertilization effect. Non-forest areas (e.g., grasslands and croplands) do not accumulate much carbon (*SI Appendix*, Fig. S5*B*) since increases in plant productivity are mainly offset by increases in respiration. The high ΔC_totecosysc_ for the high forest fraction regions indicates a strong simulated capacity of undisturbed forests to continue as a carbon sink in the future. Regions with substantial re/afforestation exhibit a somewhat lower, but still effective carbon sequestration capacity (Fig. 1*B* and *SI Appendix*, Fig. S5*C*) compared to those with intact forests, indicating that on average, forest expansion is effective in removing CO_2_ from the atmosphere.

However, re/afforestation is not uniformly effective. Notably, some regions where the Global Change Analysis Model (GCAM) suggests that forests should be planted do not show significant carbon accumulation, presumably because the future climate predicted in CESM (Community Earth System Model) does not support strong tree growth (Fig. 1 *C* and *D*). In the Central United States (CUS) and Europe (EU), for example, the re/afforested regions (*SI Appendix*, Fig. S2*I*) exhibit small or no carbon gains (Fig. 1 *A* and *C*). Specifically, the mean ΔC_totecosysc_ normalized by changes in forest cover fraction (ΔC_totecosysc_/Δ*frac_forest_*) is 0.012, 0.005, and 0.09 kg C/m^2^/% in CUS, EU, and the globe, respectively (Fig. 1*D*). In contrast, Southeast Asia (SEA), Central Africa (CAF), and South America (SAM) exhibit successful forest growth and have much larger ΔC_totecosysc_ per unit increase in forest fraction (mean ΔC_totecosysc_/Δ*frac_forest_* is 0.19, 0.28, and 0.1 kg C/m^2^/% in SEA, CAF and SAM, respectively, Fig. 1*D*). However, it is essential to recognize that there exists a dry precipitation bias in the CUS in CESM2 (31). This bias could potentially lead to an unrealistic constraint on the growth of reforested trees in that specific area.

### Differences in Carbon Emissions between the Two Alternative Land Use Pathways.

**Fig. 2.**
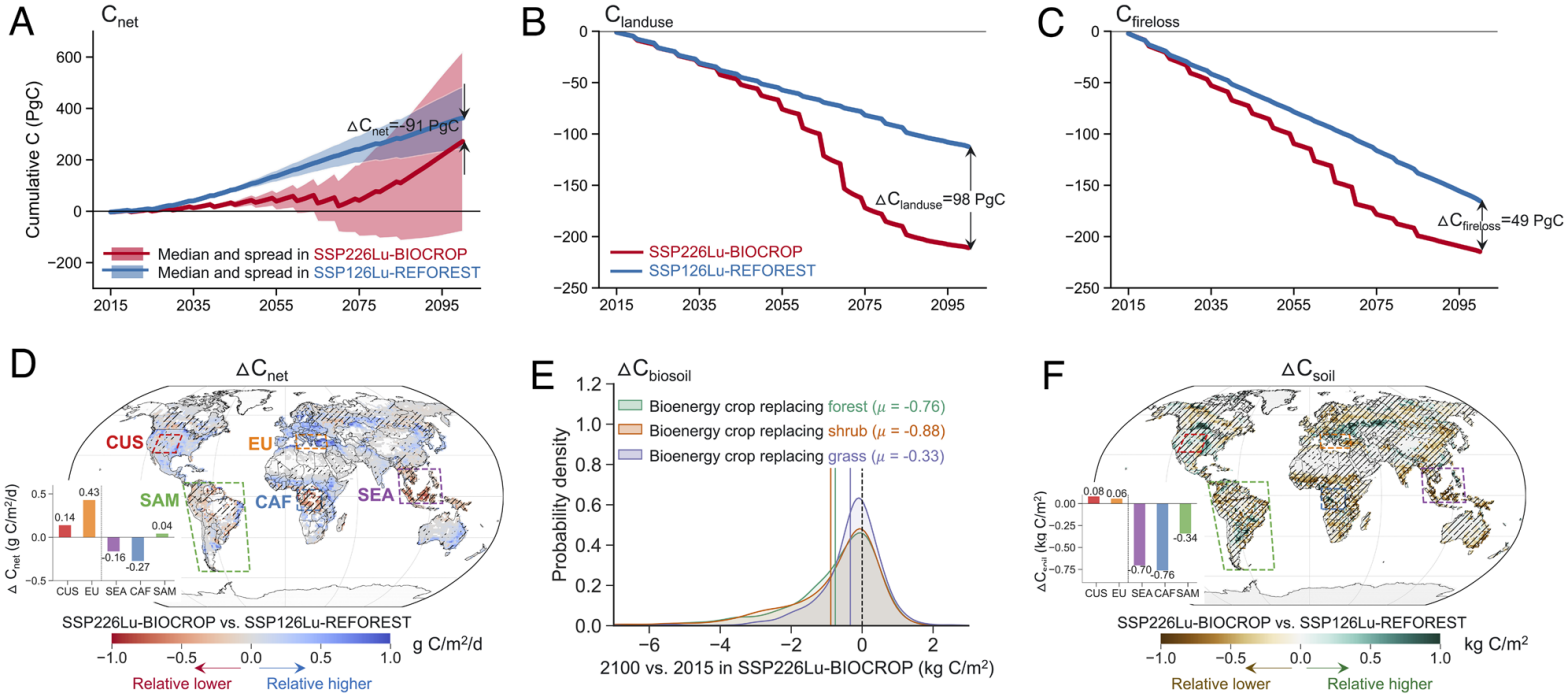
Land-use emissions associated with bioenergy expansion are responsible for lower effective land carbon sink in the bioenergy-focused scenario compared to the re/afforestation-focused scenario. Cumulative (*A*) effective land carbon sink (C_net_), (*B*) carbon emissions associated with land use change (C_landuse_), and (*C*) carbon emissions associated with fire (C_fireloss_) in the primary bioenergy expansion scenario (SSP226Lu-BIOCROP, red color) and the primary re/afforestation scenario (SSP126Lu-REFOREST, blue color) during 2015 to 2100. (*D*) Spatial differences in mean annual median C_net_ (ΔC_net_) between SSP226Lu-BIOCROP and SSP126Lu-REFOREST (SSP226Lu-BIOCROP minus SSP126Lu-REFOREST) during the end-of-the-century (2070 to 2099). (*E*) Distributions of mean annual soil carbon between 2100 and 2015 (2100 minus 2015) in SSP226Lu-BIOCROP (ΔC_biosoil_). (*F*) Spatial differences in mean annual soil carbon (ΔC_soil_) between SSP226Lu-BIOCROP and SSP126Lu-REFOREST (SSP226Lu-BIOCROP minus SSP126Lu-REFOREST) during the end-of-the-century (2070 to 2099). C_net_ comprises NBP, carbon fossil fuel offsets due to using bioenergy crops (C_offsets_), and carbon captured via BECCS (C_BECCS_). The delta values in (*A*–*C*) are SSP226Lu-BIOCROP minus SSP126Lu-REFOREST at 2100. Values of differences between the two scenarios are given for two unsuccessful forest growth regions, which are CUS (red color) and EU (orange color), and three successful forest growth regions, which are SEA (purple color), CAF (blue color), and SAM (green color) in (*D* and *F*). Positive values of C_net_, C_landuse_, and C_fireloss_ indicate carbon uptake on land, while negative values indicate carbon loss to the atmosphere. Hatches in (*D* and *F*) denote statistically significant differences at the 5% significance level.

The two alternative land use pathways have diverse impacts on land carbon sequestration (Fig. 2). The carbon emissions from land use changes associated with large-scale bioenergy crop plantation can undermine the positive effects of carbon fossil fuel offsets (C_offsets_) and BECCS (C_BECCS_) in removing carbon from the atmosphere, especially prior to 2070. By 2100, the cumulative C_offsets_ and C_BECCS_ in SSP226Lu-BIOCROP are higher than that in SSP126Lu-REFOREST by medians of 246 and 66 PgC, respectively (*SI Appendix*, Fig. S6). However, SSP126Lu-REFOREST indicates a strong effective land carbon sink (C_net_, sum of net biome production (NBP), carbon fossil fuel offsets due to bioenergy production, and carbon stored via BECCS) with a median of 363 PgC, while SSP226Lu-BIOCROP ends up with a slightly lower C_net_ with a median of 272 PgC (Fig. 2*A*). The difference is due to several reasons. First, the cumulative carbon emissions from land clearing for crop cultivation, including for bioenergy crops, is 98 PgC larger in SSP226Lu-BIOCROP than that in SSP126Lu-REFOREST (Fig. 2*B*). Second, the cumulative fire carbon loss in SSP226Lu-BIOCROP is 49 PgC higher than that in SSP126Lu-REFOREST, with tropical regions being fire hotspots due to deforestation-induced degradation fires (32) (*SI Appendix*, Fig. S7*A*). Third, there is likely a loss of “additional sink capacity” (33–35) driven by deforestation in the SSP226Lu-BIOCROP scenario (15). As noted in Fig. 1*B*, intact forests are effective carbon sinks in SSP126Lu-REFOREST, and therefore when these forests are cleared for bioenergy plantations, this constitutes a loss of sink capacity in SSP226Lu-BIOCROP.

There are large uncertainties in C_net_. While SSP126Lu-REFOREST indicates a consistent strong C_net_ with a relatively narrower range (242 to 483 PgC, Fig. 2*A*), SSP226Lu-BIOCROP exhibits a much larger spread for C_net_ (−78 to 621 PgC, Fig. 2*A*) due to uncertainties we account for in our calculations that related to future biomass yield, energy conversion technology, and CCS effectiveness. Presuming rapid and large technological advances in yield [e.g., breeding technologies (36)], land in SSP226Lu-BIOCROP could be a considerably larger effective carbon sink (by up to 138 PgC) than that in SSP126Lu-REFOREST. With slow technological advances, however, these simulations indicate that SSP226Lu-BIOCROP could fail in its intended goals and even end up being a net carbon source.

The difference in C_net_ between the two scenarios is spatially uneven and regionally dependent (Fig. 2*D*). The boreal and tropical forest areas (e.g., SEA, CAF) experience the largest reductions in C_net_ in SSP226Lu-BIOCROP relative to SSP126Lu-REFOREST. The temperate regions (e.g., CUS, EU) typically have a relatively higher C_net_ in SSP226Lu-BIOCROP (Fig. 2*D*), due to poor forest growth in these regions in SSP126Lu-REFOREST, as discussed above.

The changes in soil C depend on the original ecosystems that bioenergy crops replaced and the effectiveness of re/afforestation. In SSP226Lu-BIOCROP during 2015 to 2100, global average soil C decreases by 0.76, 0.88, and 0.33 kg C/m^2^ when bioenergy crops replace forest, shrub, or grass, respectively (Fig. 2*E*). In addition, as expected, we find that regions that are favorable for forest growth can store more soil C in SSP126Lu-REFOREST than that in SSP226Lu-BIOCROP (differences in soil C between SSP226Lu-BIOCROP and SSP126Lu-REFOREST [ΔC_soil_ = C_soil, SSP226Lu-BIOCROP_ − C_soil, SSP126Lu-REFOREST_] are −0.70, −0.76, and −0.34 kg C/m^2^ in SEA, CAF, and SAM, respectively, Fig. 2*F*). This is, however, not the case for regions with poor re/afforestation tree growth, where SSP226Lu-BIOCROP can gain a relative higher soil C than SSP126Lu-REFOREST instead (ΔC_soil_ is 0.08 and 0.06 kg C/m^2^ for CUS and EU, respectively, Fig. 2*F*).

### Differences in Regional Climate Response between the Two Alternative Land Use Pathways.

**Fig. 3.**
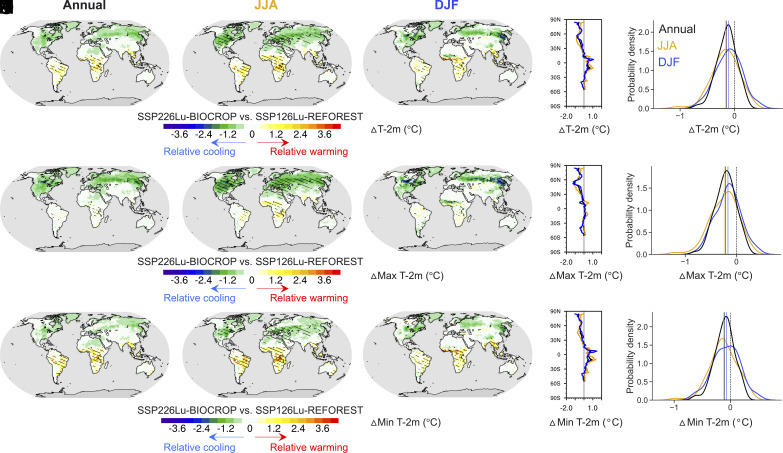
Bioenergy expansion–focused mitigation strategy can lead to a relative cooling climate compared to re/afforestation-focused mitigation strategy. Projected differences are shown for (*A*–*E*) 2-m air temperature (ΔT-2m, first row), (*F*–*J*) daily maximum of average 2-m temperature (ΔMax T-2m, second row), and (*K*–*O*) daily minimum of average 2-m temperature (ΔMin T-2m, third row) at annual (first column), June–July–August (JJA, second column), and December–January–February (DJF, third column) scales between SSP226Lu-BIOCROP and SSP126Lu-REFOREST (SSP226Lu-BIOCROP minus SSP126Lu-REFOREST) over the end-of-the-century (2070 to 2099). The plots in the fourth column (*D*, *I*, and *N*) are annual (black color) and seasonal (DJF in blue color and JJA in orange color) latitudinal-mean differences for the three variables. The plots in the fifth column (*E*, *J*, and *O*) are probability density functions of the annual (black color), JJA (orange color), and DJF (blue color) differences across the global domain. The solid vertical lines in (*E*, *J*, and *O*) indicate the respective mean value. Hatches in global maps denote statistically significant differences at the 5% significance level.

**Fig. 4.**
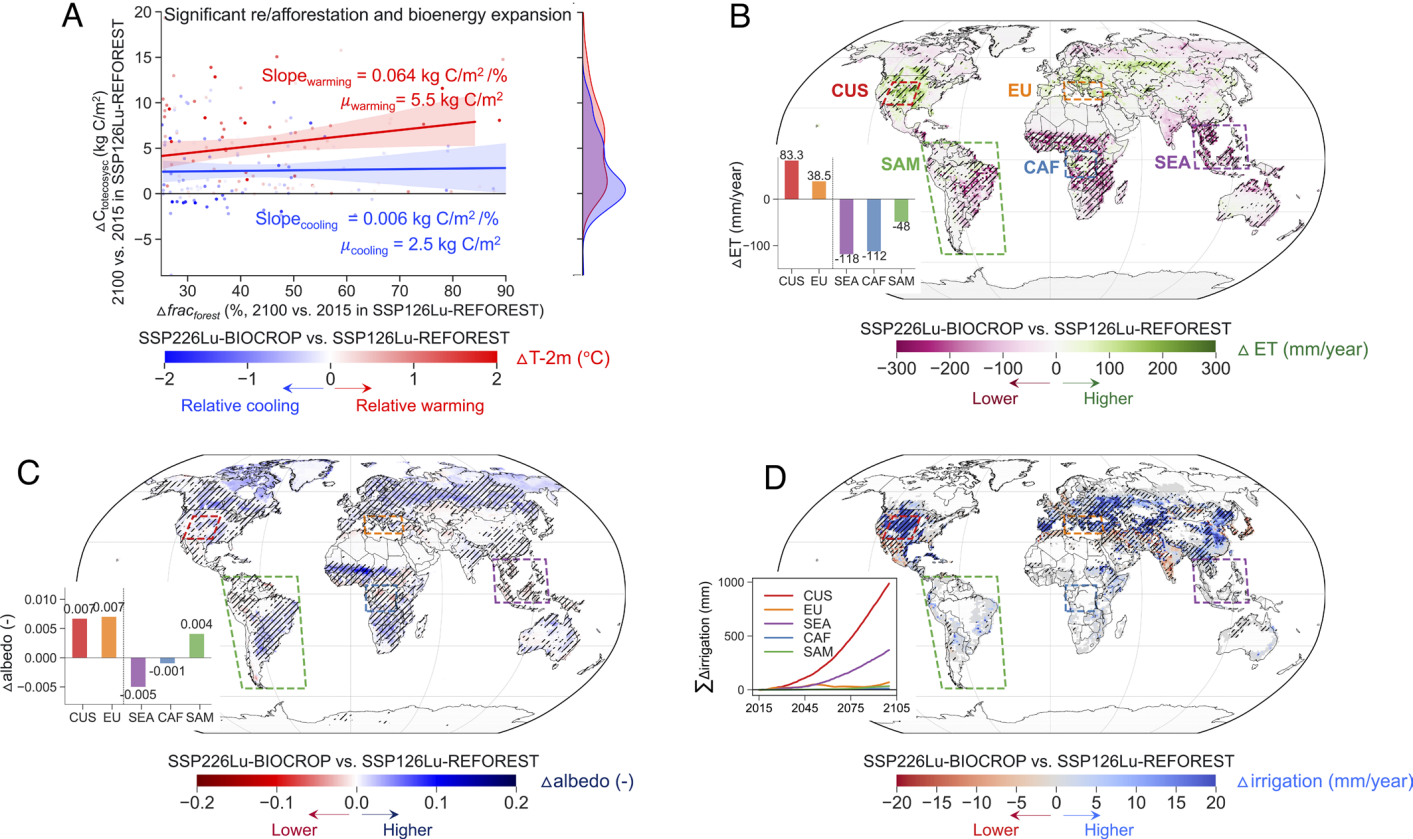
The relative summer cooling effect of SSP226Lu-BIOCROP compared to SSP126Lu-REFOREST is due to unsuccessful forest growth in certain re/afforested regions, higher albedo of bioenergy crops compared to forests, and irrigation to crops. (*A*) Scatterplot of changes in forest fraction (Δ*frac_forest_*) and changes in total ecosystem carbon (ΔC_totecosysc_) between 2100 and 2015 (2100 minus 2015) in SSP126Lu-REFOREST for the grid cells with simultaneously significant re/afforestation in SSP126Lu-REFOREST and significant bioenergy expansion in the alternative SSP226Lu-BIOCROP. The dots in (*A*) are separated based on if 2-m air temperature (T-2m) is lower (relative cooling, blue color) or higher (relative warming, red color) in SSP226Lu-BIOCROP compared to that in SSP126Lu-REFOREST (SSP226Lu-BIOCROP minus SSP126Lu-REFOREST) during summertime. The slope and μ values are average gains in C_totecosysc_ per unit increase in forest fraction and average ΔC_totecosysc_, respectively, in both the relative cooling and warming grids. The *Right* panel of (*A*) is distribution for ΔC_totecosysc_, separated by relative cooling and relative warming effects when comparing air temperature in SSP226Lu-BIOCROP with that in SSP126Lu-REFOREST (SSP226Lu-BIOCROP minus SSP126Lu-REFOREST). Spatial differences in summer (*B*) ET (ΔET), (*C*) albedo (Δalbedo), and (*D*) irrigation amount (Δirrigation) between SSP226Lu-BIOCROP and SSP126Lu-REFOREST (SSP226Lu-BIOCROP minus SSP126Lu-REFOREST) during the end-of-the-century (2070 to 2099). Values of differences between the two scenarios are given for two unsuccessful forest growth regions, which are CUS (red color) and EU (orange color), and three successful forest growth regions, which are SEA (purple color), CAF (blue color), and SAM (green color). Hatches in (*B*, *C*, and *D*) denote statistically significant differences at the 5% significance level.

We find that SSP226Lu-BIOCROP results in a global cooler climate compared to SSP126Lu-REFOREST (*SI Appendix*, Fig. S4), but the relatively cooler climate is not uniform across regions and seasons (Fig. 3 and *SI Appendix*, Figs. S8 and S9). Note that for most regions, both scenarios, as expected, experience warming over the 21st century (*SI Appendix*, Fig. S8) due to the radiative forcing associated with greenhouse gas emissions for this RCP2.6 scenario; hereafter the relative cooling/warming that we describe refers to the relative 2 m-air temperature cooling/warming in SSP226Lu-BIOCROP compared to SSP126Lu-REFOREST during the end-of-the-century. The relative cooling effect is more pronounced at high latitudes than in tropical and temperate regions (Fig. 3 *A*–*D* and *SI Appendix*, Fig. S9). In the polar zones, the annual mean air temperature in SSP226Lu-BIOCROP is cooler by up to 0.96 °C during March-April-May (*SI Appendix*, Fig. S9*C*), likely due to snow-vegetation albedo feedback (23) (*SI Appendix*, Fig. S10*A*). A relative warming in SSP226Lu-BIOCROP is seen in tropical regions across all seasons (0.18 ~ 0.39 °C, *SI Appendix*, Fig. S9) due to lower ET in SSP226Lu-BIOCROP than that in SSP126Lu-REFOREST in these regions (Fig. 4*B*). These results are in line with existing findings that croplands are generally cooler than forests in high latitudes in winter due to the snow-vegetation albedo feedback (*SI Appendix*, Fig. S10*B*) and forests are normally cooler all year around in tropical regions (23).

The two mitigation strategies also have different impacts on maximum and minimum daily temperature (Fig. 3 and *SI Appendix*, Fig. S9). When comparing average maximum and minimum daily 2-m air temperature in SSP226Lu-BIOCROP with that in SSP126Lu-REFOREST, boreal regions show the strongest relative cooling signal (Fig. 3 *F*–*O* and *SI Appendix*, Fig. S9). Specifically, the largest decreases in average maximum (1.17 °C) and minimum (0.77 °C) daily air temperature in SSP226Lu-BIOCROP compared to SSP126Lu-REFOREST occur in the northernmost latitudes (*SI Appendix*, Fig. S9*C*). Tropical regions, on the other hand, experience up to +0.14/0.48 °C increase in average maximum/minimum summer daily temperature in SSP226Lu-BIOCROP compared to that in SSP126Lu-REFOREST (*SI Appendix*, Fig. S9*D*).

### Anatomy of the Relative Cooling Effect between the Two Alternative Land Use Pathways.

The relative summer cooling effect of SSP226Lu-BIOCROP compared to SSP126Lu-REFOREST is driven by the combined effects of albedo (i.e., higher albedo of bioenergy crops compared to forests) and irrigation (i.e., irrigation for food crops) (Fig. 4). This relative summer cooling is reinforced in some re/afforested regions due to the forest health effect. This is because the local simulated environmental conditions are not conducive to tree growth, leading to relatively poor growth of re/afforested trees. Thereby, the anticipated cooling effect from forests due to higher ET does not manifest itself in these regions. Instead, the higher albedo (Fig. 4*C*) and irrigation-induced ET (23, 24) (Fig. 4*D*) in SSP226Lu-BIOCROP drives a slightly counterintuitive relative cooling in this scenario.

To demonstrate this forest health effect more directly, we focus on regions with concurrently increased bioenergy crops in SSP226Lu-BIOCROP and increased tree cover in SSP126Lu-REFOREST (*SI Appendix*, Fig. S3*D*). We find that pixels showing strong total ecosystem carbon gains associated with re/afforestation, i.e., more successful forest growth (ΔC_totecosysc_ = 5.5 kg C/m^2^), tend to experience a relative local warming climate in SSP226Lu-BIOCROP compared to SSP126Lu-REFOREST. In addition, the gain in C_totecosysc_ per unit increase in forest fraction (i.e., slope in Fig. 4*A*) is more effective in the relative warming grids (Slope_warming_ = 0.064 kg C/m^2^/%) than grids that experience relative cooling (Slope_cooling_ = 0.006 kg C/m^2^/%) (Fig. 4*A*).

This forest health effect can be further explained when comparing differences in ET (ΔET = ET_SSP226Lu-BIOCROP_ – ET_SSP126Lu-REFOREST_) between the two scenarios in regions with unsuccessful and successful reforested tree growth (see *Methods* for definition). In the two unsuccessful tree growth regions (CUS and EU), ΔET is 83.3 and 38.5 mm/y in CUS and EU, respectively, while in the two regions showcasing successful forest growth (SEA and CAF), ΔET is −118 and −112 mm/y in SEA and CAF, respectively (Fig. 4*B*). As a result, the two unsuccessful forest growth regions experience a relative local cooling climate (the differences in air temperature between SSP226Lu-BIOCROP and SSP126Lu-REFOREST [ΔT-2m = T-2m_SSP226Lu-BIOCROP_ – T-2m_SSP126Lu-REFOREST_] are −0.62 and −0.49 °C in CUS and EU, respectively) and the three successful forest growth regions experience a relative local warming climate (ΔT-2m is 0.43, 0.82, and 0.27 °C in SEA, CAF, and SAM, respectively, *SI Appendix*, Fig. S9*A*). These values are comparable to the background warming from the radiative forcing of RCP2.6 in SSP226Lu-BIOCROP (i.e., the overall change in T-2m between 2015 and 2100), which are 1.3, 0.2, 1.3, 1.4, and 1.0 °C in CUS, EU, SEA, CAF, and SAM, respectively, further demonstrating the substantial impact of land use changes. It is noteworthy that in SEA and CAF, SSP126Lu-REFOREST also exhibits a higher albedo compared to SSP226Lu-BIOCROP (Fig. 4*C*). This distinction may be attributable to the impact of exposure of dark and moist tropical soils after harvest in crop areas, which affects the regional mean albedo. This accentuates the benefits of re/afforestation strategies in tropical regions.

Across boreal regions, the relative cooling effect of SSP226Lu-BIOCROP compared to SSP126Lu-REFOREST (Fig. 3) appears related to decreased LAI (*SI Appendix*, Fig. S7*B*) and higher albedo (Fig. 4*C*) of bioenergy crops versus forests (albedo effect). Crop irrigation amplifies the relative cooling effect in the bioenergy scenario which sees more increases in food crop area (irrigation effect, Fig. 4*D*). By 2100, the cumulative differences in irrigation amount between the two scenarios are 369 mm and 11 mm for SEA and CAF, respectively. Although these two regions with successful tree growth both experience a relative warming climate in SSP226Lu-BIOCROP than that in SSP126Lu-REFOREST, the relative warming effect is attenuated in SEA relative to CAF, due to the higher irrigation application in SEA (Fig. 4*D*). This further confirms that the relative cooling in SSP226Lu-BIOCROP can be attributed to different drivers (forest health, albedo, and irrigation effects). These results imply that only if re/afforestation is situated in climates that are amenable to tree growth will the additional cooling benefits of forests be realized.

Note that the cropland area in SSP226Lu-BIOCROP is higher than that in SSP126Lu-REFOREST (*SI Appendix*, Fig. S1*E*). This explains why SSP226Lu-BIOCROP requires higher total irrigation applications (Fig. 4*D*). It is therefore essential to account for the irrigation effect stemming from these differences when considering the relative cooling impact of SSP226Lu-BIOCROP compared to SSP126Lu-REFOREST. In addition, although irrigation contributes to evaporative cooling in SSP226Lu-BIOCROP, compared to SSP126Lu-REFOREST, it comes at the cost of consuming an additional 372 km^3^ of water per year in order to meet the global irrigation demand by 2100. This could potentially worsen water stress at local scales (18). Due to the lack of additional control simulations, our study does not directly attribute the relative importance of forest health, irrigation, and albedo effects on the relative cooling impact. This could be addressed in future studies by conducting additional model simulations using time-invariant patterns of disturbed fraction (37). This approach maintains a consistent disturbed fraction pattern across simulations, which can effectively isolate the effects of specific variables of interest while minimizing the impact of other factors.

## Discussion

Despite the significance of alternative land-use pathways in meeting low climate goals under SSPs, it remains unclear whether they can achieve the intended biogeochemical and biogeophysical outcomes. Our study advances current understanding of impacts of two land-based mitigation strategies (bioenergy expansion–focused and re/afforestation-focused) in two ways. First, this study directly tests whether the end-of-the-century climate conditions can still be achieved with similar magnitude under alternative land use pathways. Second, we use a fully coupled global ESM rather than a land-only regional model, which allows us to investigate the two-way regional-to-global scale land-climate feedback that is largely overlooked in prior assessments about alternative land use pathways to meet low climate targets.

We find that the effective carbon sink (C_net_) associated with land-based mitigation solutions is strongly dependent on assumptions related to BECCS technological progress and advancements as well as on the ability of environmental conditions to support forest growth in projected re/afforested regions. This results in a wide variability of C_net_ in SSP226Lu-BIOCROP, ranging from a strong carbon sink to a weak carbon source. This also leads to substantial spatial heterogeneity in the relative difference of C_net_ between the two scenarios, with tropical/temperate regions having lower/higher C_net_ in SSP226Lu-BIOCROP than that in SSP126Lu-REFOREST. These findings underscore the importance of considering technological advances and local conditions when designing effective land-based mitigation strategies.

The two alternative mitigation scenarios also lead to spatially and seasonally distinct climate consequences (38). The maximum relative cooling of SSP226Lu-BIOCROP against SSP126Lu-REFOREST occurs in summertime over high latitudes. This is because the high-albedo-driven cooling benefit is stronger than the low-ET-driven warming contribution when comparing the two scenarios, consistent with previous findings that the forest albedo effect is more dominant at high latitudes (2, 23). In contrast, the bioenergy-driven deforestation in tropical regions causes a relative warming effect when comparing SSP226Lu-BIOCROP to SSP126Lu-REFOREST. With anticipated large increases in population (39) in the world’s most vulnerable regions (e.g., SEA, Africa, South and Central America) (40), bioenergy expansion in the tropics could potentially disproportionally expose more people to higher maximum daily temperature, despite the global intended mitigation benefits. These diverse biogeochemical and biogeophysical consequences between SSP226Lu-BIOCROP and SSP126Lu-REFOREST deserve further attention as they suggest that the SSP framework to use different land use pathways to achieve the same climate mitigation goal may not always deliver the intended benefits and that even if the global climate targets are met, regional climate differences due to local to regional land use changes can in some cases offset the mitigation targets.

While the analysis for the two scenarios used in this study primarily focuses on the differences between re/afforestation and bioenergy crop cultivation, it is important to recognize that real-world scenarios may foster the coexistence of these strategies alongside various other land-based mitigation approaches. Hence, it is essential to consider integrating these strategies in light of research findings within real-world land use practices. Notably, our study reveals variable re/afforestation effectiveness across temperate regions (Fig. 1), implying the possibility of synergistically integrating re/afforestation and bioenergy expansion to maximize climate mitigation outcomes. For instance, areas with limited forest growth potential could serve as focal points for bioenergy crop development, while tropical zones, which have substantial re/afforestation benefits, could have constrained bioenergy expansion. Our findings highlight the critical importance of optimizing the location of re/afforestation and bioenergy expansion regions in future land use planning to maximize the probability of successfully achieving the intended mitigation outcomes (23).

It should be noted that a few factors not considered in this study may affect our findings and therefore deserve more future investigations. First, due to the complexity of agricultural practices and land use change policies, it is not possible for GCAM to capture all details related to bioenergy expansion in real-world settings. Therefore, the GCAM-developed scenarios are unconstrained (see “*Limitations of GCAM*” in *Methods* for more details). Second, the relative climate impacts of the two scenarios are likely to be dependent in part on the quality of the CESM-simulated climate (see “*Simulations*” in *Methods* for more details). Third, while CESM/CLM reasonably captures the present-day CO_2_ fertilization effect (41) and reasonably replicates the present-day fire distribution, it is imperative to acknowledge that there are inherent uncertainties in the simulation of forest growth. For example, the saturation point of the fertilization effect remains uncertain, as does the influence of forest disturbances such as fires, beetles, and wind throw. Moreover, uncertainties encompass both the accuracy of forest resilience modeling and how forests respond to a changing climate, especially extreme events such as droughts. This underscores the rationale for either a multi-model ensemble approach or an ensemble focusing on structural or parametric uncertainties within a single model, in order to provide a more robust assessment of the suitability of different mitigation options. Additionally, this supports the rationale within the CLM development community for the ongoing transition from CESM/CLM to CESM/CLM-FATES (42), to enable more mechanistic representation of forest growth and disturbances. Fourth, achieving carbon mitigation benefits through the expansion of bioenergy depends on several factors, such as land-use change emissions resulting from bioenergy and food crop area expansion, carbon fossil fuel offsets, and carbon captured via BECCS. However, inconsistencies have been identified between the estimates provided by CESM and the projections generated by GCAM. Our study shows that CESM-based estimates of carbon loss driven by land-use changes (*SI Appendix*, Fig. S11*A*) are considerably higher than those from GCAM projections (*SI Appendix*, Fig. S11*B*). Specifically, by 2100, CESM estimates a cumulative land-use change emission of 211 PgC in SSP226Lu-BIOCROP (*SI Appendix*, Fig. S11*A*), while GCAM only projects 34 PgC (*SI Appendix*, Fig. S11*B*). Furthermore, the median CESM-simulated C_offsets_ and C_BECCS_ values in SSP226Lu-BIOCROP are 274 and 76 PgC lower, respectively, than the corresponding GCAM estimates (*SI Appendix*, Fig. S6). These differences are driven by several compounding model differences between GCAM and CESM (e.g., spatial resolution, biosphere representation, and assumptions for technological advances) (see “*Estimates of C Terms in CESM*” in *Methods* for more details). These discrepancies likely contribute to the carbon trajectory differences between the two scenarios that are designed to achieve the same radiative forcing by the end of the century. It is worth noting that the divergence between the two SSPs is primarily driven by underlying socioeconomic assumptions, such as changes in population growth and energy demand. These assumptions, to some extent, dictate the possible future land-use pathways. To directly and more robustly attribute land use change effects from other socio-economic factors, it is crucial to develop a set of contrasting land-use change scenarios within the same SSP family that achieve the same end-of-the-century RCP levels in the next generation of CMIPs (CMIP7).

## Methods

### Models.

To jointly examine the effects of different land use pathways (e.g., bioenergy expansion–focused and re/afforestation-focused) on biogeochemical and biogeophysical outcomes, we apply an integrated human–Earth system modeling framework called GCAM-Demeter-CESM_bioenergy_. This modeling framework uses self-consistent assumptions to develop future societal pathways (10) and consists of explicit representation of perennial bioenergy crops, including their biophysical and biogeochemical characteristics as well as agricultural management practices (29).

GCAM is an integrated assessment model (IAM) that simulates the behavior of, and interactions between energy demands, water demands, agriculture and land use, the economy, and climate (27). GCAM consists of energy, water, land, socioeconomics, and climate systems. GCAM projects future land use scenarios (2015 to 2100) at a 5-y interval. This 5-y time step is used for market prices to be adjusted and the supply and demand of goods and services to remain equilibrium (27). The CO_2_ sinks in GCAM are the amount of carbon uptake by vegetation, and the emissions include fossil fuel and industrial CO_2_, land-use change CO_2_, and carbon capture and storage CO_2_. GCAM includes the use of a generic high-yield bioenergy crop (e.g., switchgrass). The demand and supply of bioenergy are determined by the energy and land systems, respectively. GCAM accounts for the uptake of carbon during bioenergy crop growth, and the release of carbon during bioenergy combustion. Without the use of Carbon Capture and Storage (CCS), this combination of carbon uptake and release results in net zero emissions. If CCS is used, land-based emissions become net negative. Note that GCAM does not assume that bioenergy is produced from biomass removed to clear land.

Demeter is a land use and land cover change downscaling model (28, 43). Because the original land use data projected by GCAM are typically at a combination of economic region and water basin levels, ranging from a few hundreds to millions of km^2^, it is not suitable for use in gridded ESMs. Demeter disaggregates land use and land cover projections from GCAM to a spatial scale that can be directly used by ESMs (e.g., 0.05°).

CESM (Community ESM) is a widely used, fully coupled, global ESM that simulates the states of the coupled Earth system, including land, atmosphere, ocean, and sea ice (31). CESM_bioenergy_ is the CESM version with explicit representation of two perennial bioenergy crops (i.e., switchgrass and *Miscanthus*) (29, 44). The second-generation bioenergy crops are better alternatives to traditional first-generation bioenergy crops (e.g., corn and soybean) because of their higher productivity and water use efficiency as well as lower demands for irrigation and fertilization. In this study, land-use trajectories projected by GCAM and downscaled by Demeter are used as input for CESM_bioenergy_, and the two-way feedback between GCAM and CESM_bioenergy_ are not considered. While simulating re/afforestation, in areas that fail to generate a healthy forest in a future climate, since CESM in the standard configuration does not have a full Dynamic Vegetation Model component, these subgrid areas will continue to be considered as a tree plant functional type, but with stunted simulated canopy height and low vegetation carbon accumulation. However, it is important to note that in practical scenarios, inadequate tree growth could prompt a natural transition to grassland or intentional conversion to cropland, both of which would likely have a higher albedo compared to the stunted tree simulation in CESM. Notably, CESM-simulated summer albedo of stunted forests in SSP126Lu-REFOREST is lower than that of crops in SSP226Lu-BIOCROP (*SI Appendix*, Fig. S12), for 100% and 81% of the stunted forest pixels in CUS and EU, respectively. As a result, the resulting relative warming of SSP126Lu-REFOREST relative to SSP226Lu-BIOCROP, as discussed in the section “*Anatomy of the Relative Cooling Effect between the Two Alternative Land Use Pathways*,” could potentially be overestimated. Additionally, while bioenergy production can result in both CO_2_ and non-CO_2_ emissions (e.g., N_2_O emissions from fertilizer application), CESM does not reliably simulate N_2_O emissions from agricultural systems and therefore cannot currently be used to evaluate the potential impacts of different forms of land use on N_2_O emissions.

### Agricultural Management Practices in CESM_bioenergy_.

CESM_bioenergy_ has a crop module with explicit and interactive agricultural management practices, such as planting, harvesting, irrigation, and fertilization (45). These land management practices are applied to all crops, including perennial bioenergy crops. Perennial bioenergy crops have a longer growing season than annual crops and their harvesting date is once per year during the late winter. About 70% of the aboveground biomass (leaves and stems) of perennial bioenergy crops is removed at the harvest time step and put into a 1-y biomass product pool that gradually decays. This harvested carbon is assumed to be used for BECCS, thereby offsetting carbon emissions that would have otherwise arisen from using fossil fuels. The food crops are both rain-fed and irrigated, whereas the bioenergy crops are simulated as rain-fed only. Areas with irrigated food crops are projected by GCAM (46) and subsequently downscaled by Demeter (10). Irrigation is applied for these food crops when the available soil water content falls below predetermined soil moisture thresholds (45, 47, 48). It should be noted that all crop areas, including bioenergy crops and food crops, are implicitly no-till in CESM. In reality, tilling practices could lead to additional soil carbon losses (49), especially in land-demanding low-emission scenarios that involve substantial bioenergy and food crop expansions.

### Future Land Use and Land Cover Change Scenarios.

Two future scenarios (SSP226Lu-BIOCROP and SSP126Lu-REFOREST) with contrasting land use pathways to achieve the same 2.6-W/m^2^ radiative forcing by 2100 are examined (*SI Appendix*, Fig. S2 and Table S1). The two land use pathways are a primary bioenergy expansion pathway and a primary re/afforestation pathway. Different IAMs may use different underlying socioeconomic and policy assumptions when simulating future scenario projections (50). In this study, the primary bioenergy expansion (SSP226Lu-BIOCROP) and the primary re/afforestation (SSP126Lu-REFOREST) scenarios are co-developed using the same IAM—GCAM, thereby minimizing the impact of socio-economic uncertainties that are IAM-dependent. Note that establishing switchgrass from native grassland likely requires tillage, which can lead to a substantial carbon debt. In contrast, re/afforestation generally incurs a lower carbon cost during establishment. GCAM considers such carbon costs associated with various land uses when making overall decisions regarding land use application in different regions (51). Consequently, in an SSP1 world, we incentivize re/afforestation due to the values of carbon in the land system. In contrast, in SSP2, we incentivize low-carbon energy systems, allowing the land system to adapt to growing energy crops at the expense of land use changes. We are using these scenarios as a means to a) primarily understand the consequences of a scenario that is dominated by re/afforestation versus one that is dominated by bioenergy expansion, b) but more generally to evaluate possible climate consequences of two different SSPs that meet the same radiative forcing target (i.e., RCP2.6).

Between 2015 and 2100 (2100 minus 2015), GCAM projects approximately 17.6 million km^2^ global land areas of bioenergy crops in the primary bioenergy expansion scenario (SSP226Lu-BIOCROP) and 10.7 million km^2^ global re/afforested regions in the primary re/afforestation scenario (SSP126Lu-REFOREST, *SI Appendix*, Fig. S1). The bioenergy expansion in SSP226Lu-BIOCROP is accompanied with 9.8, 5.0, and 6.2 million km^2^ decreases in areas of forest, shrub, and grass, respectively, while the forest expansion in SSP126Lu-REFOREST is accompanied with 4.6, 6.3, and 6.3 million km^2^ decreases in areas of shrub, grass, and crop, respectively. Note that food crop areas also increase by 2.5 million km^2^ in SSP226Lu-BIOCROP (*SI Appendix*, Fig. S1*E*) to meet a growing food demand of rising population. In contrast, the relatively low population along with assumed declining preferences for meat and high agricultural yields in SSP1 lead to shrinking cropland in SSP126Lu-REFOREST (*SI Appendix*, Fig. S1*E*). The land use data is at a 5-y frequency, which means the land cover information is the same during 2015 to 2019, 2020 to 2024, etc. This drives the 5-y periodicity of the effective land carbon uptake, as seen in Fig. 2 *A*–*C*. Note that SSP126Lu-REFOREST also has bioenergy expansion (5.8 million km^2^), about a third as much as that in the bioenergy scenario (*SI Appendix*, Fig. S1*A*), as the deployment of BECCS occurs in both scenarios in GCAM. Therefore, SSP226Lu-BIOCROP is a primarily bioenergy expansion scenario and SSP126Lu-REFOREST is a primarily re/afforestation scenario as it is a mix of bioenergy expansion and re/afforestation. These two scenarios also include localized deforestation (*SI Appendix*, Fig. S2 *D* and *I*). For simplicity, this paper refers to these two scenarios as SSP226Lu-BIOCROP and SSP126Lu-REFOREST, even though the actual scenarios are more complex than that. It should also be noted that among all IAMs used in AR6, GCAM projects the highest bioenergy development and the bioenergy crop area for 2100 used in our study (17.6 million km^2^) exceeds even the upper bound (10.9 million km^2^) outlined in AR6 (8). This discrepancy arises primarily from different versions of GCAM (AR6 uses GCAM v4.2 while our study employs GCAM v4.3). Given the computational-intensive nature of the Demeter downscaling process, high-resolution downscaled land use data are only available from this specific GCAM version (GCAM v4.3).

### Simulations.

In this study, we run CESM_bioenergy_ at a 0.9° × 1.25° spatial resolution. Two concentration-driven numerical experiments (SSP226Lu-BIOCROP and SSP126Lu-REFOREST) are conducted, each with three ensemble members to account for internal variability and uncertainty in the climate system (*SI Appendix*, Table S1). Note that we do not find substantial differences in the results among the ensembles, especially for carbon variables. As such, we present the ensemble-mean results throughout the manuscript.

Given the complexity of biophysical effects of land use changes on climate, it is difficult to fully establish the underlying physical mechanisms that drive responses to land-use change in different parts of the world. In the context of this single model study, we show that the climate responses due to land-use change in these scenarios are consistent with changes in albedo and ET, though clearly other factors like aerodynamic roughness and atmospheric feedback related to changes in circulation or clouds will also play a role.

Uncertainties surrounding the effectiveness of re/afforestation should be considered, which depends on the existence, strength, and duration of the CO_2_ fertilization effect. For example, tentatively explained by a hypothetical saturation of the CO_2_ fertilization, field-based evidence suggests that intact Amazon forest could become a carbon source to the atmosphere in about 15 y from now (52). Current ESMs need to be improved to better capture this carbon sink saturation effect. Additionally, phosphorus availability may affect how tropical forest responds to elevated CO_2_ due to the potential limitation of nutrient on tree productivity (53, 54), which could further constrain the effectiveness of re/afforestation-based mitigation efforts.

It should also be noted that there is a dry precipitation bias in the Central U.S. region in CESM Version 2 (CESM2) (31) that may be unrealistically limiting growth of reforested trees in that region. Improved identification of what regions would be suitable for future re/afforestation practices requires either substantial bias reduction in future generation ESMs or other means to bias correct ESM output, for example, through anomaly-forced land-only simulations (55). Future work could also involve similar experiments in a multi-model context to better establish to what extent model structures and parameters could impact the results. Additional model improvement of CESM to factor in the potential annual yield increase of bioenergy crops driven by technology advances (6, 56) could lead to more realistic assessment of future water consumption of bioenergy crops as well as the associated regional climate outcomes (57).

### Estimates of Land-Use Change C Emissions in GCAM.

GCAM tracks C emissions due to land use changes separately from fossil fuel and industrial CO_2_ emissions. The land-use change C emissions are a function of changes in total land area (e.g., land expansion or land contraction) and carbon density. Land-use C emissions are allocated across a vegetation-specific time, which is related to how long it takes for the vegetation (e.g., forest or crop) to mature. The land-use emissions include vegetation (aboveground) and soil (belowground) carbon emissions. For more information about calculation of land-use change C emissions in GCAM, please refer to ref. 27.

### Estimates of C Terms in CESM.

The effective land carbon sink (C_net_, Fig. 2*A*) is the sum of NBP, carbon fossil fuel offsets (C_offsets_), and carbon uptake via BECCS (C_BECCS_). NBP is calculated as gross primary productivity minus ecosystem respiration (ER) and carbon losses due to fires and land-use changes. Positive/negative values of C_net_ indicate carbon sequestration/release on land. Specifically, C_offsets_ is the amount of carbon that is prevented from being released to the atmosphere due to the utilization of bioenergy crops for fuel instead of fossil fuels. C_offsets_ is calculated by utilizing the carbon released into the atmosphere from the 1-y harvested bioenergy product pool in CESM, along with two key parameters (i.e., fossil fuel offset ratio and the ratio of carbon content of fossil fuels to the carbon content of bioenergy crops) derived from GCAM (16). Variations in estimating C_offsets_ (*SI Appendix*, Fig. S6*A*) arise from the fact that the offsets depend on the fossil fuel offset ratio and the carbon content of different types of fossil fuels that are being displaced (e.g., natural gas, oil, or coal) (16). The C_BECCS_ value has ranges (*SI Appendix*, Fig. S6*B*) because it depends on the percentage of bioenergy used with CCS and also the CCS efficiency, both of which are uncertain (16). More details on estimates of C_net_, C_offsets_, and C_BECCS_ can be found in ref. 16.

The differences exist in the predicted C_offsets_ and C_BECCS_ between CESM and GCAM (*SI Appendix*, Fig. S6) primarily arise from how these two models simulate crops, including crop yield simulation, bioenergy crop types, crop harvest percentages, and crop yield enhancement assumptions. To elaborate, first, GCAM employs a combination of economic and biophysical relationships to predict how changes in climate, technology, and land use affect crop production. Specifically, GCAM’s crop production is a function of factors such as land competition based on yield and profitability, the influence of socioeconomics on food and energy demands, and the climate-induced effects, specifically those resulting from the climate scenario (RCP2.6 in this study), on water availability and yield responses. In contrast, CESM uses a physically-based crop model to explicitly simulate crop growth. This entails comprehensive representation of critical crop physiological processes such as photosynthesis, carbon allocation, and phenology, subject to localized climate conditions, and agricultural management practices, including planting, irrigation, fertilization, and harvesting. Second, GCAM considers diverse bioenergy crops and aggregates them into two categories, “biomass_grass” and “biomass_tree.” Consequently, the bioenergy crops within GCAM embody combined characteristics of a variety of crop types rather than resembling a specific one. However, due to data availability and model limitations, the current version of CESM only simulates switchgrass in our study. Third, GCAM assumes complete bioenergy crop harvesting for energy production, while CESM assumes that only 70% of the aboveground biomass is being harvested. Fourth, GCAM projects how future changes in agriculture and technology might affect crop yields, including the potential for enhanced yields driven by market-induced incentives and technology advancements. Specifically, the SSP226Lu-BIOCROP and SSP126Lu-REFOREST scenarios examined in this study explore the future trajectories SSP1 and SSP2. The associated agricultural enhancements outlined in each storyline could influence crop production, encompassing aspects like fertilizer application, yield advancements, and changes in irrigation technology. Conversely, the future crop yield estimated by CESM largely depends on local climatic conditions and farming practices and does not account for such feedback from market influences and technological changes.

Due to inconsistencies in simulated carbon stored via BECCS between ESM (*SI Appendix*, Fig. S6*B*) and IAM (*SI Appendix*, Fig. S6*D*), existing studies suggest that ESM-simulated C_BECCS_ by 2100 should be scaled by a factor of 2.5 to 3 to have commensurate values with that used in IAMs. This scaling factor roughly equates to a 2% annual yield improvement over the period 2015 to 2100 (2), which is also assumed by a U.S. Department of Energy report on biofuels (56) in its moderate-yield scenario. Given that future crop yield estimated from CESM is predominantly influenced by climatic conditions and does not factor in technological advances, as previously discussed, we follow this 2% annual increase to accommodate potential increases in future biomass yield.

CESM also simulates the C emissions associated with land use changes (C_landuse_, Fig. 2*B*). C_landuse_ is the sum of total C that is directly emitted from land cover conversion (C_conversion_) and C losses from the grain and wood product pools (C_productloss_). When the vegetation area contracts, CESM maintains the same carbon density for the remaining vegetation areas, which means a decrease in total remaining carbon mass given that the total area has decreased. The lost C mass is either immediately released to the atmosphere, or sent to product pools to gradually decay, or sent to litter pools in CESM. Positive and negative values of C_landuse_ indicate carbon uptake on land and carbon loss to the atmosphere, respectively. Note that there are some differences in calculating C_landuse_ between GCAM and CESM, mainly due to the methodological differences between these two models in terms of how the carbon density of crops and the amount of land converted are calculated. In GCAM, C_landuse_ is a function of changes in total land area and carbon density, and it is allocated across time based on the assumed duration required for the vegetation to mature. GCAM does not consider spatial variations and temporal dynamics when calculating C_landuse_. For example, C_landuse_ is not dependent on climate and CO_2_ impacts on vegetation carbon stocks. In CESM, components of C_landuse_ depend on the carbon stocks of the vegetation, which dynamically respond to different climate, CO_2_, and nutrient availability conditions. Differences in the simulated vegetation carbon stock or carbon density between GCAM and CESM can lead to large differences in the simulated C_landuse_. Moreover, the spatial resolutions of GCAM and CESM are different. GCAM divides the global land area into 384 regions based on 32 geopolitical regions and 235 water basins, while CESM is ran at a 0.9° × 1.25° spatial resolution. This could result in potential area differences in the amount of land converted for each crop types between GCAM and CESM as well.

The C emission associated with fire (C_fireloss_, Fig. 2*C*) is also considered in CESM. Burned area is affected by climate and weather conditions, vegetation composition and structure, and human activities. When a fire occurs, carbon is removed from the grid cell and the vegetation structure is modified accordingly. Subsequently, the burned area is not tracked going forward, and the post-fire impact is only reflected through the degraded forest with reduced carbon, LAI, and shorter vegetation. Deforestation in tropical systems can also drive degradation fires, which are escaped fires that occur when deforestation by fire extends beyond the originally intended region. The spread of degradation fires is determined by deforestation rate and climate. Boreal fires warrant significant attention in Earth system modeling due to their substantial carbon emissions and intricate interplay with the climate. To improve the simulation of boreal fires, CESM2/CLM5 restricts fire occurrence when the surface air temperature is below the freezing point, thereby improving the modeling of fire seasonality in the boreal zone. Broadly speaking, the CESM2/the Community Land Model Version 5 (CLM5) fire module produces fire statistics that are commensurate with other global ESMs (58). However, recent extreme fire behavior in the western United States and Canada, fire activity that is higher than that predicted by CESM2, raises the prospect that current generation fire models are underpredicting the rate of change in fire behavior in response to climate change. Comprehensive assessment of whether recent fire activity reflects an epochal shift in fire response to climate change or is simply a response to internal climate variability or other drivers of fire activity is beyond the scope of this paper. Nonetheless, the results from CESM2 should be viewed from the lens that the model may be underpredicting the rate of change of fire behavior, which would have additional consequences for the effectiveness of the re/afforestation and the land carbon sink. For more comprehensive details on the fire module in CESM, interested readers are referred to ref. 59 and references therein.

### Limitations of GCAM.

Our study only simulates switchgrass as a representative high-yield bioenergy crop, while sugarcane (60) and other types of second-generation bioenergy crops (22) are also used in bioenergy productions in other regions of the world. Given the growing evidence that different bioenergy crop types may result in different carbon and climate responses (e.g., surface-atmosphere energy and mass fluxes), future work need to extend the bioenergy crop family to better characterize how different crop types may result in different carbon and climate responses (22).

Socioeconomic factors associated with region-specific land use change policies deserve future attention for GCAM modeling communities. First, the hotspots of real-world bioenergy expansion do not always coincide with those in GCAM projections. For example, Latin America has been identified as a hotspot where most arable and suitable land would be available for expansion of bioenergy and food crops in coming decades (57). However, GCAM projections do not align with this analysis. Second, many bioenergy incentives schemes in the United States (e.g., the Renewable Fuel Standard) and EU [e.g., Renewable Energy Directive (RED II)] exclude bioenergy feedstock production on newly cleared forestlands, and/or try to quantify and avoid leakage (indirect land use changes). These factors are currently not considered in GCAM either. Future scenario development should pay greater attention to reducing the divergence between projected scenarios and the evolving best sustainable practices and policies. Third, there are on-the-ground socioeconomic challenges involved in large-scale land use transformation (61), such as the intricate land tenure issue in the tropical regions (62).

### Different Types of Land Use Changes.

We analyze four types of grid cells based on the land-use changes occurring in the grids: i) retain a high and constant forest fraction (*frac_forest_*) throughout 2015 to 2100, ii) retain a low and constant forest fraction throughout 2015 to 2100, iii) undergo significant re/afforestation, and iv) undergo simultaneously significant re/afforestation and bioenergy expansion. The four types of land pixels are defined in Eqs. **1**–**4**, respectively (also see *SI Appendix*, Fig. S3). In particular, to analyze grid cells with considerable re/afforestation relatively early in the 21st century, the significant re/afforestation and bioenergy expansion is defined as when the difference of *frac_forest_* or *frac_bioenergy_* between 2060 and 2015 is greater than or equal to 25% (Eqs. **3** and **4**, *SI Appendix*, Fig. S3*D*). The simultaneously significant re/afforestation and bioenergy expansion means the places are concurrently experiencing significant re/afforestation in the SSP126Lu-REFOREST scenario and significant bioenergy expansion in the alternative SSP226Lu-BIOCROP scenario (Eq. **4**).

[1] $$\begin{array}{l} frac_{forest,\;\text{SSP126Lu}-\text{REFOREST},\;2100}\;\geq \;90\%\;\text{and}\\ \Delta \;frac_{forest,\;\text{SSP126Lu}-\text{REFOREST},\;\;2100\;\;\text{v}\text{s}.\;\;2015\;}\leq \;\;5\%, \end{array}$$

[2] $$\begin{array}{l} frac_{forest,\;\text{SSP126Lu}-\text{REFOREST},\;2100}\;\leq \;10\%\;\;\text{and}\\ \Delta \;frac_{forest,\;\text{SSP126Lu}-\text{REFOREST},\;\;2100\;\;\text{v}\text{s}.\;\;2015}\leq \;\;5\%, \end{array}$$

[3] $$\begin{array}{c} \Delta frac_{forest},{\;}_{\text{SSP}126\text{Lu}-\text{REFOREST},\;2060\;\text{vs}.\;2015}\;\geq \;25\%, \end{array}$$

[4] $$\begin{array}{c} \Delta frac_{forest},{\;}_{\text{SSP}126\text{Lu}-\text{REFOREST},\;2060\;\text{vs}.\;2015}\;\geq \;25\%\;\text{and}\\ \;\Delta frac_{bioenergy},{\;}_{\text{SSP}226\text{Lu}-\text{BIOCROP},\;2060\;\text{vs}.\;2015}\;\geq \;25\%. \end{array}$$

### Successful/Unsuccessful Forest Growth Regions.

Pixels projected to undergo significant re/afforestation in SSP126Lu-REFOREST, where planted trees are simulated to grow well, are defined as regions with successful forest growth. This definition is based on the criterion that the change in total ecosystem carbon normalized by the change in forest fraction (ΔC_totecosysc_/Δ*frac_forest_*) between 2015 and 2100 (2100 minus 2015) in these regions is greater than 0.05 kg C/m^2^/%. In SSP126Lu-REFOREST, there are also pixels with projected re/afforestation but actually experience relatively stunted simulated growth of trees (i.e., ΔC_totecosysc_/Δ*frac_forest_* less than 0.05 kg C/m^2^/%) due to local environment not conducive to tree growth. These regions are defined as unsuccessful forest growth regions. In this study, two unsuccessful forest growth regions (i.e., CUS and EU) and three successful forest growth regions (i.e., SEA, CAF, and SAM) are selected for close analyses. Increases in forest areas by 2100 relative to 2015 are 0.43, 0.19, 0.15, 0.63, and 2.24 million km^2^ in CUS, EU, SEA, CAF, and SAM, respectively.

### Models and Land Use Datasets.

CESM is publicly available at https://github.com/ESCOMP/CESM. GCAM is publicly available at https://github.com/jgcri/gcam-core. The Demeter model is publicly available at https://github.com/JGCRI/demeter. The GCAM version used to create the Demeter outputs is publicly available at https://zenodo.org/record/3713432. The Demeter version used to create the downscaled land use data is publicly available at https://zenodo.org/record/3713378. The original GCAM output databases are publicly available at https://dataverse.harvard.edu/dataset.xhtml?persistentId=doi:10.7910/DVN/DYV29J. The Demeter-downscaled land use data used to drive the CESM simulations in this study are publicly available at https://doi.org/10.25584/data.2020-07.1357/1644253.

## Supplementary Material

Appendix 01 (PDF)

## Data Availability

Data and code used for analysis in this study are available at https://github.com/yychengESM/2023_Cheng_bioenergy_vs_reforestation.git (63).
