# Supplementary material for: A bioenergy-focused versus a reforestation-focused mitigation pathway yields disparate carbon storage and climate responses
Source: Proc Natl Acad Sci U S A. 2024 Feb 5;121(7):e2306775121. doi: 10.1073/pnas.2306775121 (PMC10873610; doi:10.1073/pnas.2306775121)
Supplement: Supplementary file 1 — Appendix 01 (PDF) [file pnas.2306775121.sapp.pdf]

Supplementary Information for

A bioenergy-focused versus a reforestation-focused mitigation pathway yields disparate carbon storage and climate responses

Cheng *et al*.

Table S1: Summary of the numerical experiments conducted in this study.

Name of experiment	Spatial resolution	Climate	Land use	Period	Short description
SSP226Lu-BIOCROP	0.9°×1.25°	SSP1-2.6	SSP2-2.6	2015-2100	**Primary bioenergy expansion**, the SSP2-2.6 land use is used within the SSP1-2.6 climate simulation
SSP126Lu-REFOREST	0.9°×1.25°	SSP1-2.6	SSP1-2.6	2015-2100	**Primary re/afforestation**, the SSP1-2.6 land use is used within the SSP1-2.6 climate simulation

*Each scenario was performed with three ensembles using different initial conditions.

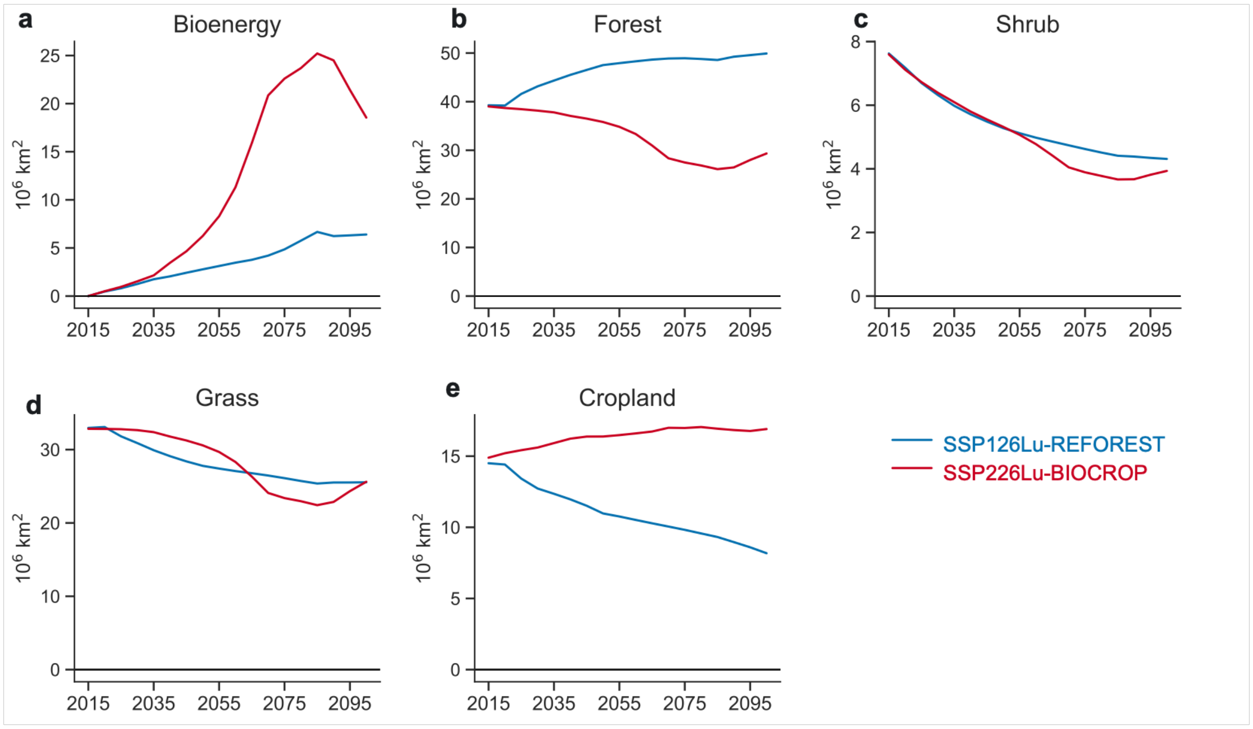

Fig. S1: Changes in plantation areas of five dominant vegetation types. Changes in total areas of (**a**) bioenergy crops, (**b**) forest, (**c**) shrub, (**d**) grass, and (**e**) cropland during 2015-2100 in the primary bioenergy expansion scenario (SSP226Lu-BIOCROP, red color) and the primary re/afforestation scenario (SSP126Lu-REFOREST, blue color) scenarios. Here the cropland in **e** includes areas of corn, soybean, wheat, cotton, rice, sugarcane, and C3 unmanaged crops.

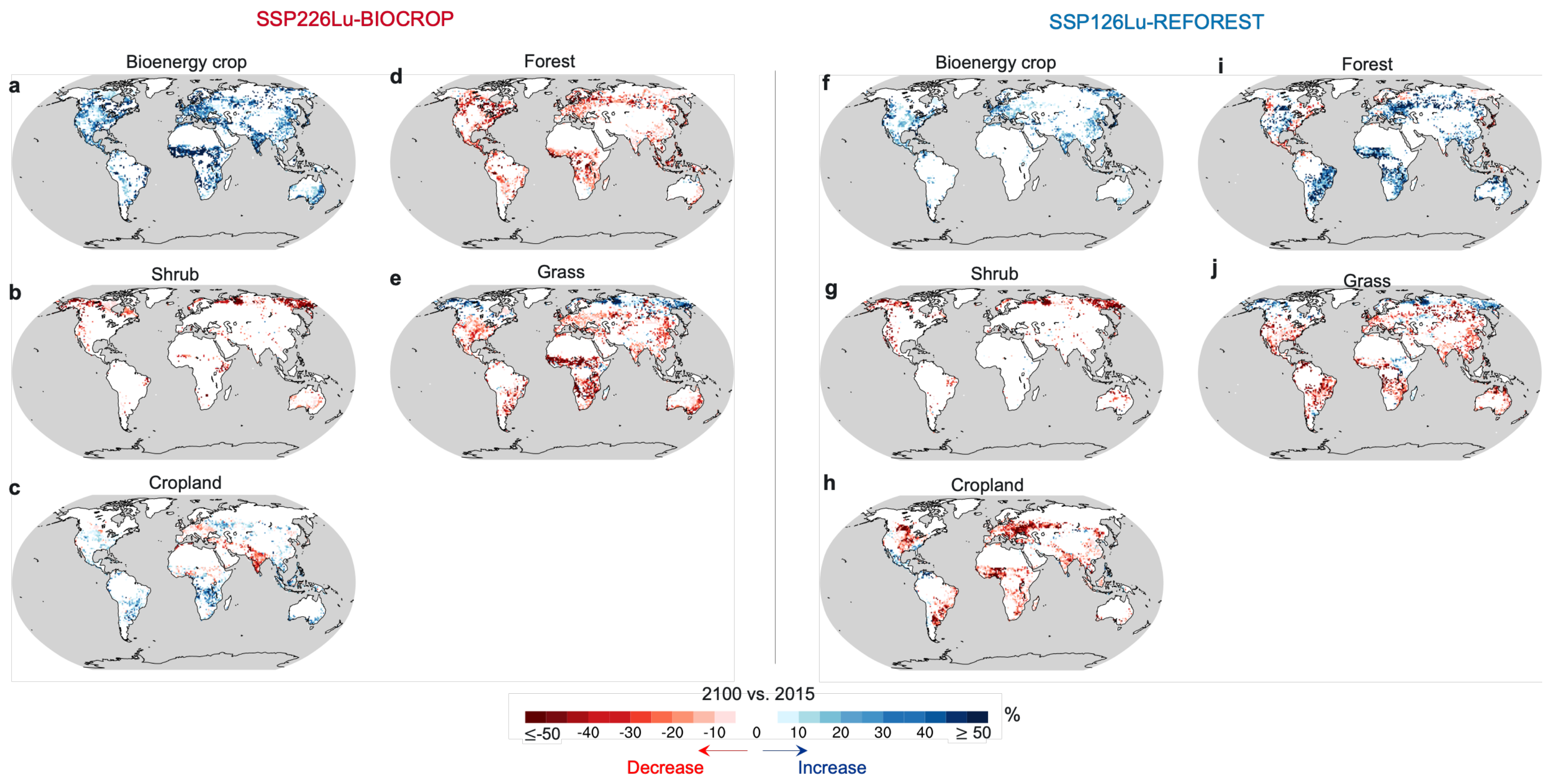

Fig. S2: Spatial fractional changes of five dominant vegetation types. Changes in percentage of (**a** and **f**) bioenergy crops, (**b** and **g**) shrub, (**c** and **h**) cropland, (**d** and **i**) forest, and (**e** and **j**) grass in SSP226Lu-BIOCROP (**a-e**, left panel) and SSP126Lu-REFOREST (**f-j**, right panel) between 2100 and 2015 (2100 minus 2015). Here the cropland in **c** and **h** includes areas of corn, soybean, wheat, cotton, rice, sugarcane, and C3 unmanaged crops.

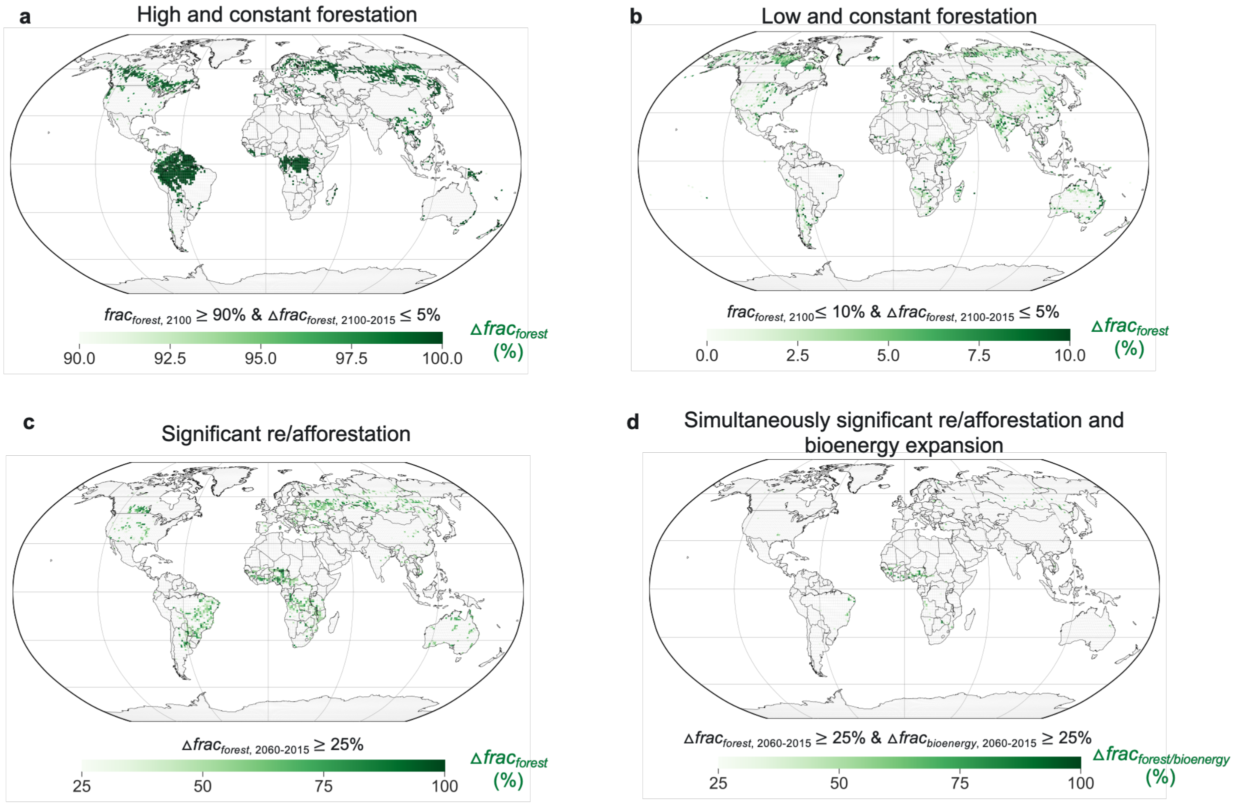

Fig. S3: Changes are shown for forest fraction ($frac_{forest}$) in 2100 for **a** grid cells with high and constant forest fraction ($frac_{forest,\ 2100} \geq 90\%$ and $\Delta frac_{forest,\ 2100\ \text{vs.}\ 2015} \leq 5\%$), **b** grid cells with low and constant forest fraction ($frac_{forest,\ 2100} \leq 10\%$ and $\Delta frac_{forest,\ 2100\ \text{vs.}\ 2015} \leq 5\%$), **c** grid cells with significant re/afforestation ($\Delta frac_{forest,\ 2060\ \text{vs.}\ 2015} \geq 25\%$), and **d** grid cells with simultaneously significant re/afforestation and bioenergy expansion ($\Delta frac_{forest,\ 2060\ \text{vs.}\ 2015} \geq 25\%$ and $\Delta frac_{bioenergy,\ 2060\ \text{vs.}\ 2015} \geq 25\%$). See more definition in Methods.

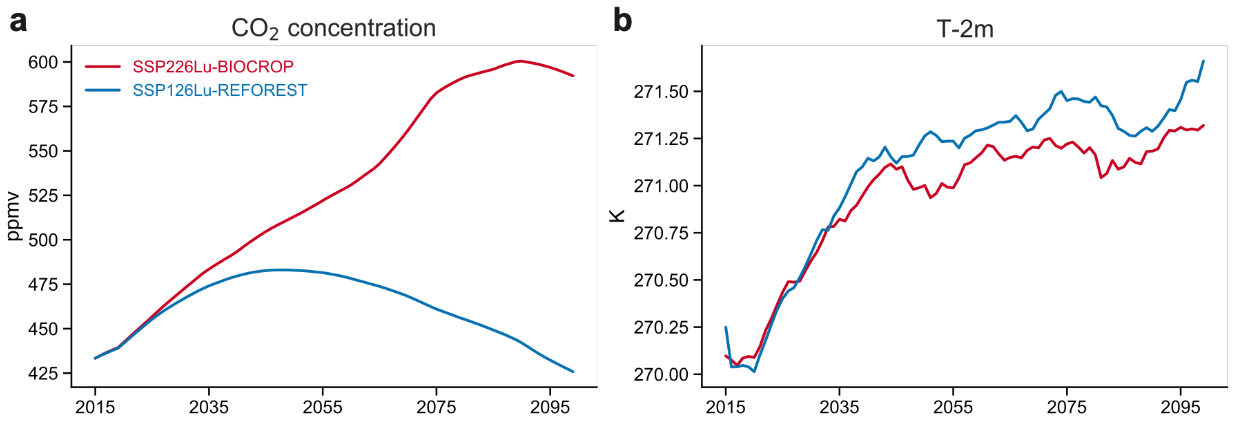

Fig. S4: Global average mean annual **a** CO_2 concentration and **b** 2-m air temperature (T-2m**)** from 2015-2100 in the primary bioenergy expansion scenario (SSP226Lu-BIOCROP, red color) and the primary re/afforestation scenario (SSP126Lu-REFOREST, blue color).

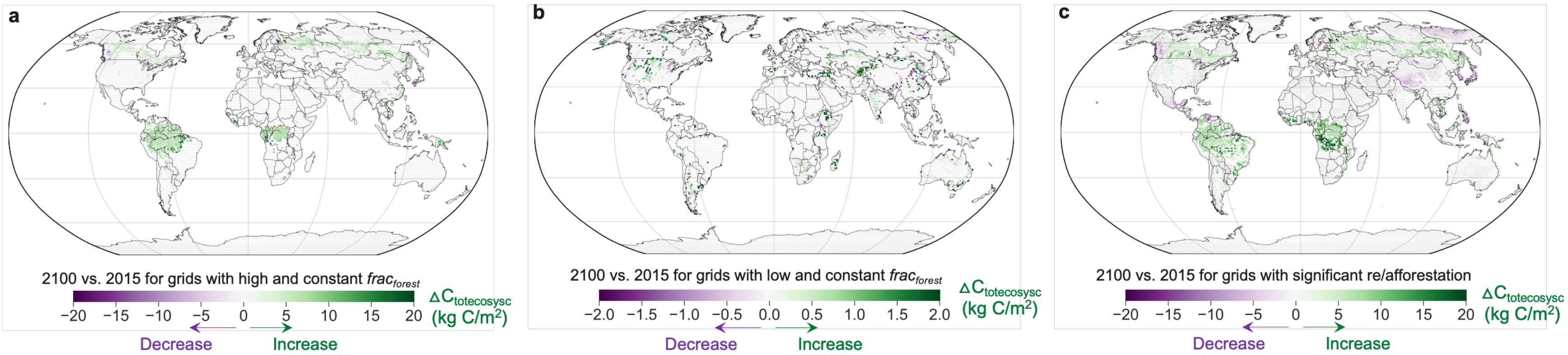

Fig. S5: Changes in total ecosystem carbon between 2100 and 2015 in the primary re/afforestation scenario. Changes are shown between 2100 and 2015 (2100 minus 2015) for total ecosystem carbon ($\Delta C_{totecosysc}$) for **a** grid cells with high and constant forest fraction ($frac_{forest,\,2100} \geq 90\%$ and $\Delta frac_{forest} \leq 5\%$), **b** grid cells with low and constant forest fraction ($frac_{forest,\,2100} \leq 10\%$ and $\Delta frac_{forest} \leq 5\%$), and **c** grid cells with significant re/afforestation ($\Delta frac_{forest,\,2060\text{ vs. }2015} \geq 25\%$). See more definition of high/low and constant forest fraction and significant re/afforestation in Methods.

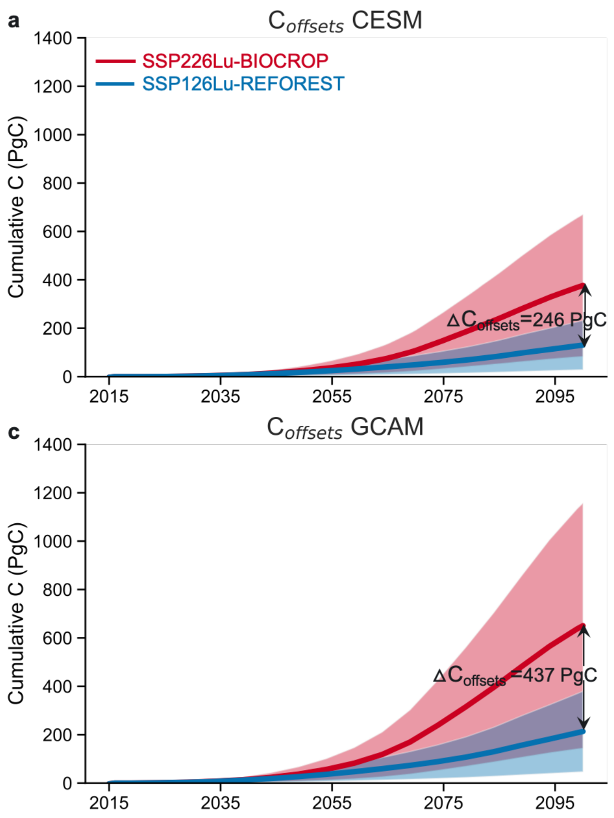

Fig. S6: CESM- and GCAM-simulated carbon fossil fuel offsets and carbon captured via BECCS in the primary bioenergy expansion scenario and the primary re/afforestation scenario. a Carbon fossil fuel offsets due to using biofuels instead of fossil fuels ($C_{offsets}$) and **b** carbon captured via BECCS (C_{BECCS}) simulated by CESM, **c** $C_{offsets}$ and **d** C_{BECCS} simulated by GCAM in the primary bioenergy expansion scenario (SSP226Lu-BIOCROP, red color) and the primary re/afforestation scenario (SSP126Lu-REFOREST, blue color) during 2015-2100.

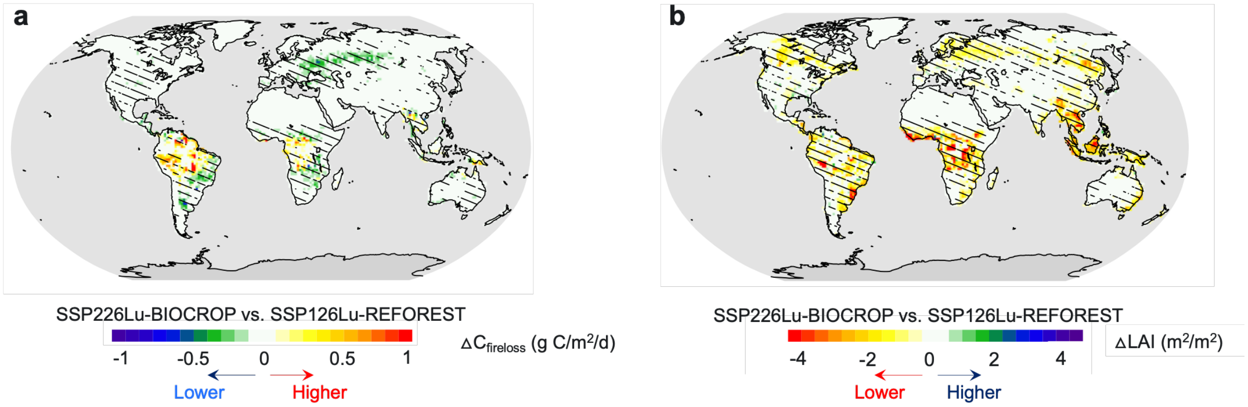

Fig. S7: Projected changes for mean annual **a** carbon loss due to fire ($\Delta C_{fireloss}$) and **b** leaf area index (ΔLAI) over the end-of-the-century (2070-2099) between SSP226Lu-BIOCROP and SSP126Lu-REFOREST. Hatches denote statistically significant differences at the 5% significance level.

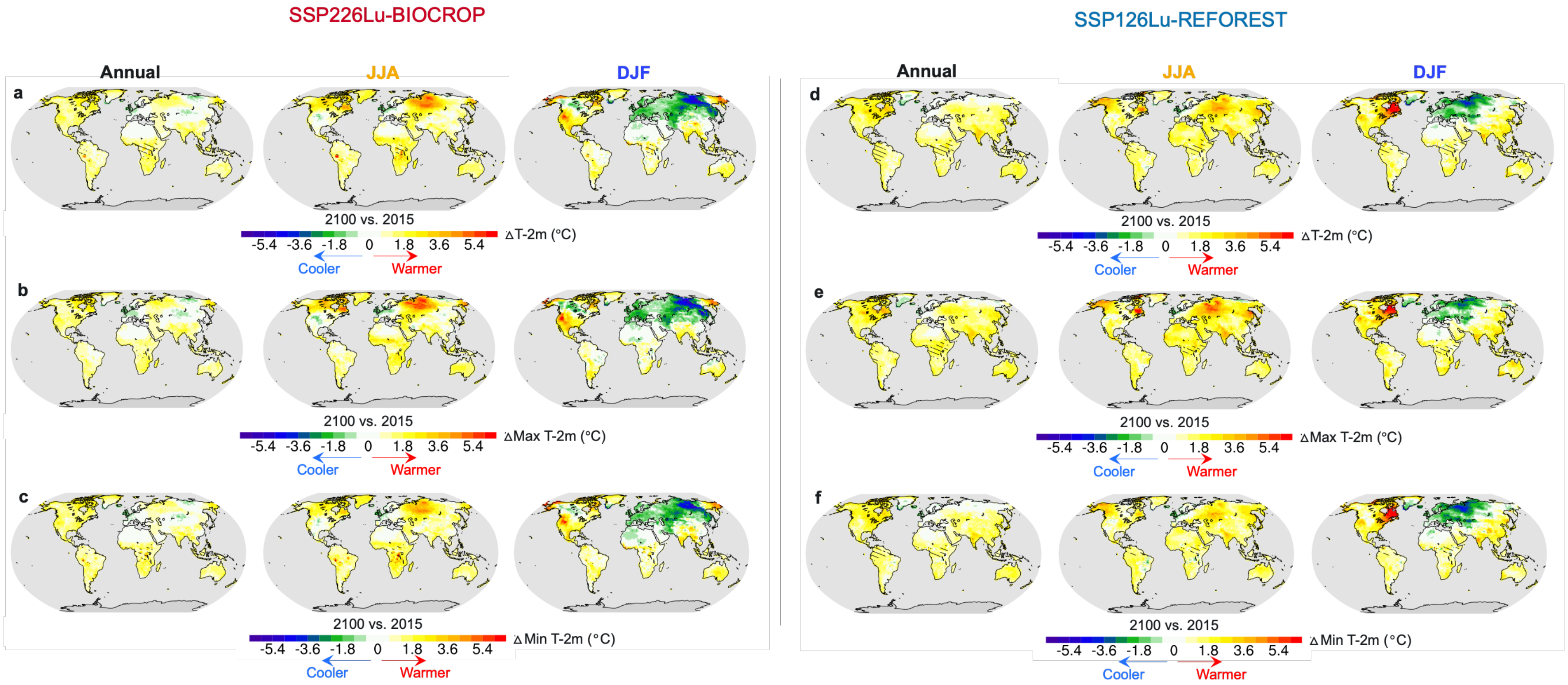

Fig. S8: Changes in temperature between 2100 and 2015 for the primary bioenergy expansion scenario and the primary re/afforestation scenario. Changes are shown for **a** and **d** 2-m air temperature (T-2m, first row), **b** and **e** daily maximum of average 2-m temperature (Max T-2m, second row), and **c** and **f** daily minimum of average 2-m temperature (Min T-2m, third row) at annual (first column), June-July-August (JJA, second column), and December-January-February (DJF, third column) scales between 2100 and 2015 (2100 minus 2015) in the primary bioenergy expansion scenario (SSP226Lu-BIOCROP, left panel) and the primary re/afforestation scenario (SSP126Lu-REFOREST, right panel). Hatches in global maps denote statistically significant changes at the 5% significance level.

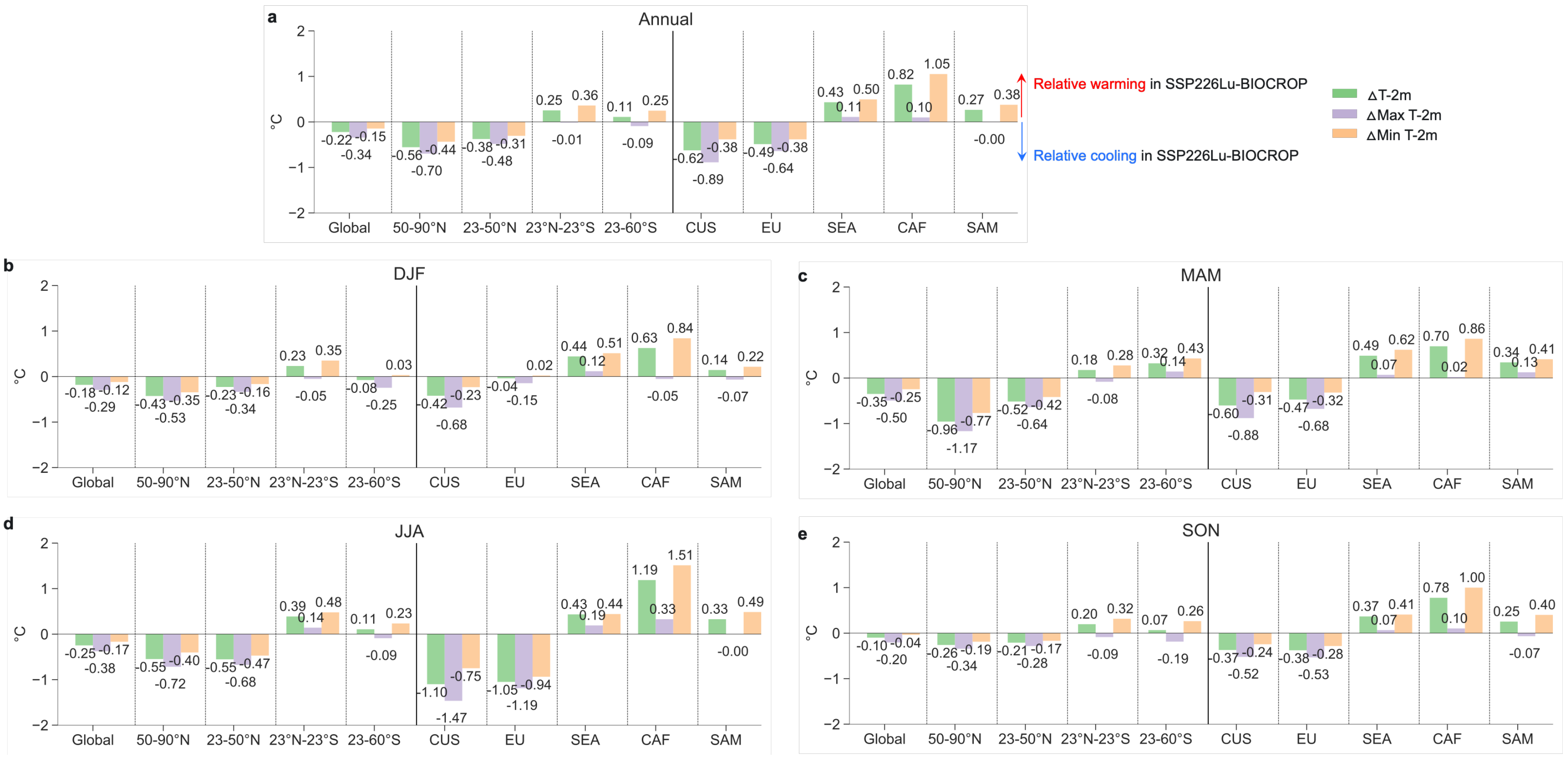

Fig. S9: Difference in air temperature between the primary bioenergy expansion scenario and the primary re/afforestation scenario during the end-of-the-century. Changes in 2-m air temperature (T-2m, green color), daily maximum of average 2-m temperature (Max T-2m, purple color), and daily minimum of average 2-m temperature (Min T-2m, yellow color) at **a** annual, **b** DJF, **c** MAM, **d** JJA, and **e** SON scales for five latitudinal zones, two unsuccessful forest growth regions (Central US [CUS] and Europe [EU]), and three successful forest growth regions (Southeast Asia [SEA], Central Africa [CAF], and South America [SAM]). The numbers are for differences between the primary bioenergy expansion (SSP226Lu-BIOCROP) and the primary re/afforestation (SSP126Lu-REFOREST) scenarios (SSP226Lu-BIOCROP minus SSP126Lu-REFOREST) over the end-of-the-century (2070-2099).

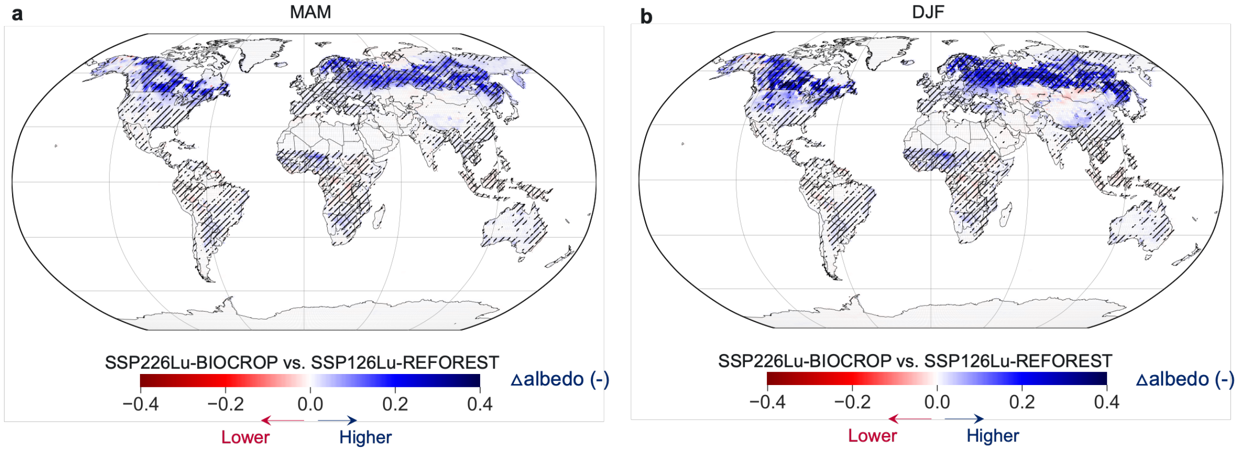

Fig. S10: Spatial differences in albedo between SSP226Lu-BIOCROP and SSP126Lu-REFOERST (Δalbedo, SSP226Lu-BIOCROP minus SSP126Lu-REFOREST) during the end-of-the-century (2070-2099) averaged over **a** March-April-May (MAM) and **b** December-January-February (DJF) time. Hatches in global maps denote statistically significant changes at the 5% significance level.

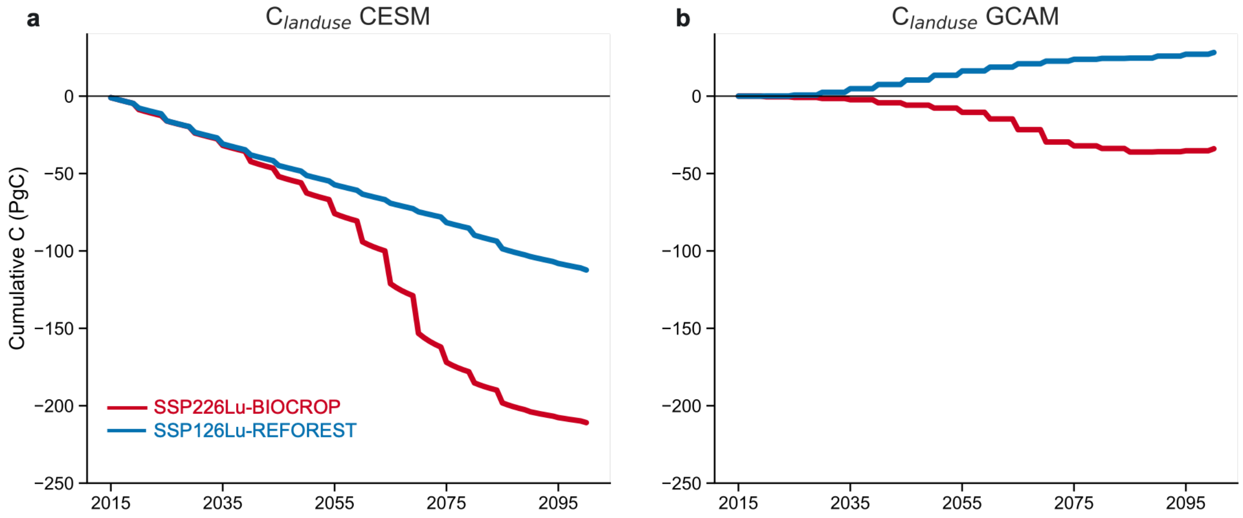

Fig. S11: Cumulative carbon emissions associated with land use change ($C_{landuse}$) simulated by **a** CESM and **b** GCAM in the primary bioenergy expansion scenario (SSP226Lu-BIOCROP, red color) and the primary re/afforestation scenario (SSP126Lu-REFOREST, blue color). Positive and negative values of $C_{landuse}$ indicate carbon uptake on land and carbon loss to the atmosphere, respectively.

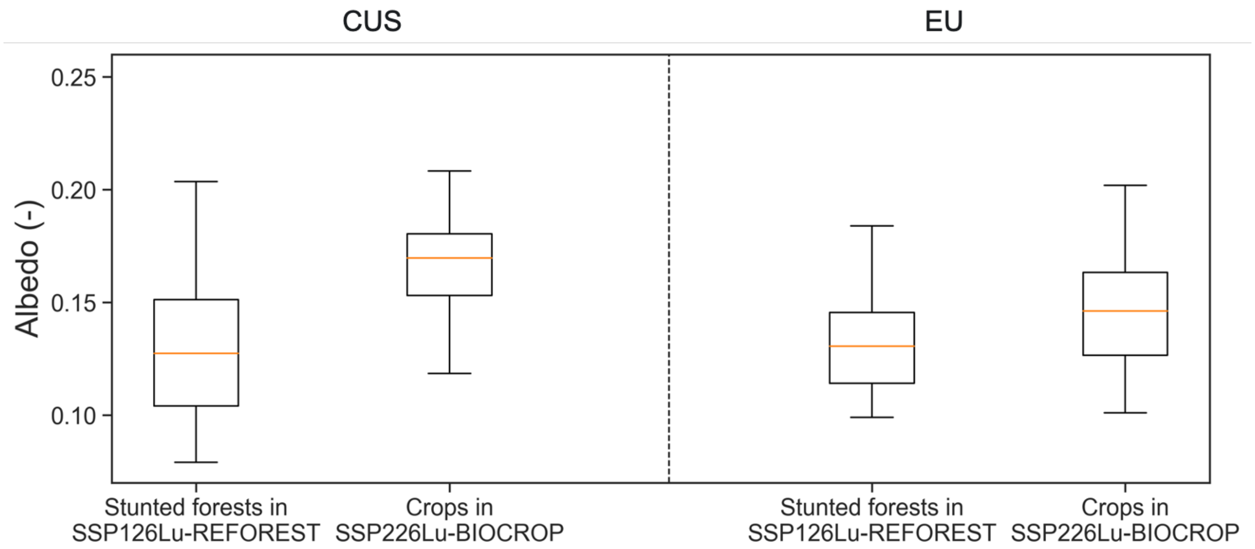

Fig. S12: Comparison of the simulated summer albedo for stunted forests in SSP126Lu-REFOREST and that for crops in SSP226Lu-BIOCROP in Central U.S. (CUS) and Europe (EU).
